# Diversity, chemical constituents and biological activities of endophytic fungi from *Alisma orientale* (Sam.) Juzep.

**DOI:** 10.3389/fmicb.2023.1190624

**Published:** 2023-06-21

**Authors:** Nayu Shen, Zhao Chen, GuiXin Cheng, Wenjie Lin, Yihan Qin, Yirong Xiao, Hui Chen, Zizhong Tang, Qingfeng Li, Ming Yuan, Tongliang Bu

**Affiliations:** ^1^College of Life Sciences, Sichuan Agricultural University, Ya’an, China; ^2^Ya’an People’s Hospital, Ya’an, China; ^3^Sichuan Agricultural University Hospital, Ya’an, China

**Keywords:** endophytic fungi, diversity, antioxidant, bacteriostatic activity, *Alisma orientale* (Sam.) Juzep., chemical constituents, high-throughput sequencing

## Abstract

The dried tuber of *Alisma orientale* (Sam.) Juzep. (AOJ) is a traditional Chinese medicine with high medicinal value. The endophytic fungi of medicinal plants are a treasure house of natural compounds. However, there is a lack of research on the diversity and biological activity of endophytic fungi of AOJ. In this study, high-throughput sequencing technology was used to study the diversity of endophytic fungi in the roots and stems of AOJ, and endophytic fungi with a high output of phenols and flavonoids were screened by chromogenic reaction, and the antioxidant and antibacterial activities and chemical constituents of crude extracts of their fermentation broth were studied. A total of 3,426 amplicon sequence variants (ASVs) belonging to 9 phyla, 27 classes, 64 orders, 152 families, and 277 genera were identified from AOJ. There were significant differences in the endophytic fungal communities of AOJ roots and stems, as well as in the endophytic fungal communities of triangular AOJ and circular AOJ. In addition, 31 strains of endophytic fungi were isolated from AOJ, of which 6 strains had good antioxidant and antibacterial activities. The crude extract of YG-2 had the strongest free radical scavenging ability and bacteriostatic ability, and its IC_50_ *_*DPPH*_*, IC_50_ *_*ABTS*_*, and IC_50_*_⋅*OH*_* values were 0.009 ± 0.000 mg/mL, 0.023 ± 0.002 mg/mL, and 0.081 ± 0.006 mg/mL, respectively. The results of LC-MS showed that the main component of the crude extract of YG-2 was caffeic acid (10.12 μmol/g). Overall, the results of this study preliminarily elucidated the diversity and community composition of endophytic fungi of AOJ, indicating that AOJ endophytic fungi have abundant secondary metabolites and good antioxidant and antibacterial activities. This study provides an important reference for further research, development and utilization of AOJ endophytic fungi and a theoretical basis for the further development of the endophytic fungus YG-2 (*Chaetomium globosum*) as a source of antioxidants.

## 1. Introduction

Due to factors such as respiration and radiation, free radicals are constantly produced in the body. The presence of a large number of free radicals will destroy the body’s equilibrium, resulting in oxidative stress and thus damaging biomolecules (Sharma et al., 2018). Current research results show that oxygen free radicals are directly related to most diseases in the human body (Marx, 1986; Moskovitz et al., 2002; Tappel and Tappel, 2004; Tang et al., 2022). Therefore, the search for effective antioxidants has become very important research. The resistance of pathogenic bacteria to existing drugs has become a global concern, and it is urgent to find new natural effective antimicrobials (Dos Santos et al., 2015; Gu et al., 2022).

Endophytic fungi live in plants and exist in mutually beneficial symbiosis with their hosts. They can’t only significantly affect the formation of metabolites in their hosts (Jia et al., 2016) but also produce the same metabolites as their hosts (Gupta et al., 2020; Li et al., 2022). Endophytes are important sources of active natural products capable of biosynthesizing medically important “phytochemicals” that were originally thought to be produced only by the host plant, and as alternative and sustainable sources, endophytes are able to produce these compounds more quickly than plants (Wen et al., 2022). Most of the active compounds isolated from endophytic fungi have antibacterial activity, and mycelium and fermentation liquid extracts can produce certain inhibitory activity against plant, animal and human pathogens (Charria-Girón et al., 2021; Gu et al., 2022). Some secondary metabolites of endophytic fungi have good antibacterial activity and have been studied for the development of new drugs (Atiphasaworn et al., 2017; Mookherjee et al., 2020; Muthukrishnan et al., 2022; Wen et al., 2022). The endophytic fungi isolated from most plants, especially medicinal plants, showed excellent antioxidant activity. Endophytic fungi isolated from medicinal plants can produce abundant bioactive secondary metabolites, such as alkaloids, terpenoids, steroids, flavonoids, quinones, isocoumarins, lignans, phenylpropanes, phenols, and lactones (Dos Santos et al., 2015; Tang et al., 2021). Moreover, these active ingredients have corresponding rich biological functions, such as antioxidant, antibacterial, antiviral, and antidiabetic activities (Muthukrishnan et al., 2022). To date, endophytic fungi with antioxidant or antibacterial activities have been isolated from medicinal plants, such as *Loranthus tanakae* Franch. and Sav, *Passiflora incarnata*, *Ocimum basilicum*, *Ligusticum chuanxiong* Hort, and *Radix Puerariae*, and many studies have shown that phenols and flavonoids rich in medicinal plants are important sources of antioxidant or antibacterial ability of endophytic fungi of medicinal plants (Atiphasaworn et al., 2017; Silva et al., 2020; Tang et al., 2021; Deng et al., 2022; Zheng et al., 2022). Zheng et al. (2022) isolated 8 endophytic fungi that produce flavonoids from the medicinal plant *Mulberry parasitica*, among which the antioxidant and antibacterial activities of *Streptomyces* ZP28 and ZM148 were higher than those of the other 6 strains. The results of LC-MS showed that the structural types of flavonoids in the extracts of ZP28 and ZM148 were mainly flavonoids and isoflavones. Li et al. (2022) isolated endophytic fungi from *Andrographis* and determined the total phenol content and total flavonoid content of strains with strong antioxidant capacity. The results showed that the extracellular extracts of the endophytic fungi AP-1 and AP-4 showed strong antioxidant activity, and the extracellular extracts of AP-1 and AP-4 had total phenol contents of 110.194 ± 11.800 μg and 101.576 ± 9.073 μg GAE/mL, respectively, And the total flavonoid contents were 87.217 ± 8.854 μg and 77.101 ± 3.510 μg RE/mL, respectively. The extraction of active ingredients from natural medicinal plants is low in efficiency, consumes plant resources and is not friendly to the ecology, while microbial fermentation has many advantages, such as easy control conditions and a short cycle time (Silva et al., 2020). The use of endophytic fungi to develop medicinal plant chemical constituents with high economic value has become a research hotspot in agriculture, medicine, industry and other fields (Ding et al., 2018; Deng et al., 2022; Zheng et al., 2022). The diversity of endophytic fungi is considered to be an important factor affecting plant productivity and health, and the diversity of the endophytic microbial community is related to plant types and organs (Wu et al., 2022). As a kind of microbial resource with broad application prospects, the diversity of plant endophytic fungi is worth exploring (Fan M. et al., 2020; Fan et al., 2022).

*Alisma orientale* (Sam.) Juzep. (AOJ) is a perennial herb of Alismataceae. The dried tuber of AOJ has been widely used in traditional Chinese medicine clinical compounds and Chinese patent medicine (Kim et al., 2016; Zhao et al., 2018; Feng et al., 2021). This plant has many pharmacological activities, such as diuresis and lowering blood pressure and blood lipids (Zhang L. et al., 2017; Xu et al., 2019). AOJ is rich in bioactive ingredients, such as polysaccharides, triterpenes, flavonoids, and alkaloids, which have been proven to have anti-inflammatory, antitumor, antioxidant and other activities (Zhang L. et al., 2017; Xu et al., 2019; Feng et al., 2021). AOJ is widely distributed, mainly in Sichuan, Fujian, Jiangxi, Guangxi, etc. In addition, AOJ has an annual production of approximately 8000 tons, which represents over 90% of the national total (Xin et al., 2017; Liu et al., 2020). Overall, AOJ has high economic and medicinal value. At present, research on AOJ focuses on its water extract, alcohol extract and terpenoid compounds, and research on its diuresis, anti-calculi and hypolipemia is more adequate (Liu et al., 2020). It has been reported that AOJ contains flavonoids and phenolic acids (Huang et al., 2017; Zhang L. et al., 2017). Phenolic substances and flavonoids have been proven to have strong antioxidant and antibacterial effects in several studies. However, there are few reports on AOJ phenols and flavonoids. In addition, studies on the antioxidant and antibacterial activity of AOJ are still insufficient. Moreover, as a widely used Chinese medicine, there are few reports on AOJ endophytic fungi.

Therefore, in this study, high-throughput sequencing technology and traditional culture methods were used to study the diversity of AOJ root and stem endophytic fungi. Phenols and flavonoids were used as screening criteria to obtain AOJ endophytic fungi with antioxidant and antibacterial activities. The chemical composition and antioxidant and antibacterial activities of extracts from the fermentation broth of endophytic fungi producing phenols and flavonoids were studied. This study is helpful to supplement the insufficient research on AOJ phenols and flavonoids, contribute to the preliminary description of the community diversity of AOJ endophytic fungi, contribute to the development and utilization of AOJ endophytic fungi resources, and provide a theoretical basis for the development of a new way to produce natural antioxidants and antibacterial drugs.

## 2. Materials and methods

### 2.1. Experimental materials

**FIGURE 1:**
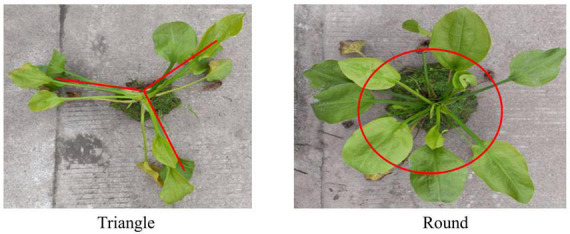
Two kinds of *Alisma orientale* (Sam.) Juzep.

As shown in Figure 1, the stems of AOJ grow into two shapes: round and triangular. The roots and stems of AOJ that grew into triangles were denoted as SG and SJ, respectively. The roots and stems that grew into rounded AOJ were denoted as YG and YJ, respectively. Fresh whole AOJ plants were collected from a farm at Sichuan Agricultural University (Ya’ an City, Sichuan Province, China). All samples were sent to the laboratory for testing immediately after collection.

### 2.2. High-throughput sequencing

After the samples were washed, the tissues were disinfected on the surface, frozen with liquid nitrogen, and transported to Beijing Novogene Technology Co., Ltd. on dry ice for high-throughput sequencing analysis in the ITS1-1F region (internal transcribed spacer-1-1F). Three samples were taken from each of the two kinds of AOJ roots and stems, for a total of 12 samples: Z1, three repeated round AOJ roots; Z2, three repeated round AOJ stems; Z3, three repeated triangle AOJ roots; and Z4, three repeated triangle AOJ stems.

### 2.3. Isolation, identification, and phylogenetic analysis of endophytic fungi

Tissue surface disinfection was improved based on the experimental methods of Rehman et al. (2022) and Sohrabi et al. (2023). The stems and roots of AOJ were rinsed with clean water for 4 h, blotted with filter paper, and transferred to a clean workbench. The AOJ tissue was washed in sterile water for 30 s, soaked in 75% ethanol for 3 min, washed in sterile water for 30 s, soaked in 2% NaClO (root for 3 min, stem for 5 min), washed in sterile water for 30 s, washed in 75% ethanol again for 30 s, washed in sterile water for 30 s, and then drained with sterile filter paper. The stems were peeled and cut into 0.5 cm^3^ chunks. The skin of the root was gently scraped off with a blade and cut into small 1 cm pieces. The cut tissue pieces were placed on potato dextrose agar (PDA) medium containing 50 μg/L ampicillin sodium and kanamycin sulfate. PDA plates were cultured in an incubator at 28°C for 7–10 days. The isolated strains were stored in the Laboratory of Molecular Biology and Biochemistry, College of Life Science, Sichuan Agricultural University. The isolated endophytic fungi were sequenced by morphology and DNA sequencing, the morphology of endophytic fungi on solid medium was observed, and the morphological characteristics of the mycelia of endophytic fungi were observed by a CX23 microscope (Olympus, Tokyo, Japan). The genomic DNA of endophytic fungi was obtained by the CTAB method, and the ITS region (internal transcribed spacer region) was amplified by primers ITS1 (TCCGTAGGTGAACCTGCGG) and ITS4 (TCCTCCGCTTATTGATATGC). The amplification conditions were the same as those of Tang et al. (2021), and the PCR products were sent to Tsingke Biotechnology Co., Ltd. for sequencing. The sequencing results were compared by BLASTN, and MEGA6 software was used to construct phylogenetic trees by the neighbor joining method.

### 2.4. Screening of endophytic fungi producing phenols and flavonoids

Mycelia of each purified fungus were selected and placed in a 250 mL triangular bottle containing 100 mL potato dextrose broth (PDB) medium and cultured at 28°C and 120 rpm for 7 days. Whatman No. 1 filter paper was used to separate the mixture by vacuum filtration to obtain filtrate (Rehman et al., 2022). The obtained filtrate was used as a test sample, and a color reaction was used for preliminary screening of the endophytic fungi producing flavonoids and phenols. The filtrate reacted with 0.1% FeCl_3_:0.1% K_3_[Fe(CN)_6_] = 1:1 solution reaction (blue solution indicates it contains phenols), 1% FeCl_3_ solution reaction (wine-red solution indicates it contains phenols) and 1 mol/L NaOH solution reaction (yellow, orange, or red solution indicates it contains flavonols).

### 2.5. Preparation of extracts of fermentation broth of endophytic fungi

Endophytic fungi producing phenols or flavonoids were expanded for fermentation using the culture method mentioned in Section “2.4. Screening of endophytic fungi producing phenols and flavonoids.” The experimental method was modified on the basis of Tang et al. (2020) and Bora and Devi (2023), four layers of gauze were used to filter the fermentation solution, collect the filtrate, extract it three times with an equal volume of ethyl acetate, collect the ethyl acetate part, and concentrate it to 1/4 of the original volume at 45°C by rotating evaporation. After freezing-drying, the fermentation solution extract was obtained, and 200 mg/mL of the fermentation solution extract was prepared by adding dimethyl sulfolone. The samples were stored at 4°C for later use.

### 2.6. Determination of the total phenol content (TPC) and total flavonoid content (TFC)

The Folin–Ciocalteu (FC) method used to determine the TPC of endophytic fungal extracts was modified from the method of Minussi et al. (2003), Li et al. (2022), and Wairata et al. (2022). A standard solution of gallic acid (0.0–0.10 mg/mL) was prepared accurately with gallic acid as the standard substance. Deionized water (200 μL) was added to the EP tube in addition to 200 μL gallic acid solution and 100 μL Folinal phenol reagent. After mixing, the solution was allowed to stand for 4 min, and 200 μL of 5% Na_2_CO_3_ solution was added. The absorbance of the samples was measured at 760 nm after the samples were diluted to 1 mL with deionized water and reacted at room temperature for 30 min. The linear regression equation, *y* = 14.303x + 0.0905 (*R*^2^ = 0.9965), was obtained by drawing the standard curve with the mass concentration (x) and absorbance (y) of the gallic acid solution. The fungal extract solution was diluted with deionized water (0.1 mg/mL), and the absorbance of the sample at 760 nm was obtained according to the steps of the standard curve. The linear regression equation was used to calculate the mass concentration of TPC in the sample solution (mg/mL). TPC is expressed in milligrams of gallic acid per gram of crude extract, i.e., mg GAE/g.

The standard curve of flavonoids was drawn by the aluminum nitrate colorimetric method (Chen et al., 2020; Zhang et al., 2023). The standard solution of rutin was 0.1 mg/mL after accurately weighing 5 mg rutin and using 70% ethanol solution to a constant volume of 50 mL. Precision suction standard solutions (0.0, 0.1, 0.2, 0.3, 0.4, and 0.5 mL) were placed in six 1.5 mL EP tubes and supplemented with 30% ethanol to 0.5 mL. Then, 30 μL 5% sodium nitrite solution was added, mixed well and allowed to stand for 6 min. Then, 30 μL 10% aluminum nitrate solution was added, mixed and allowed to stand for 6 min. Then, 0.4 mL of 4% sodium hydroxide solution was added, filled to 1 mL with deionized water, and reacted at room temperature for 15 min, and finally the absorbance was measured at 510 nm. The linear regression equation, *y* = 2.3019x + 0.0025 (*R*^2^ = 0.9988) was obtained by drawing a standard curve based on the mass concentration (x) and absorbance (y) of the rutin solution. The fungal extract solution (0.1 mg/mL) was diluted with deionized water, and the absorbance of the samples at 510 nm was obtained according to the steps of the standard curve. The linear regression equation was used to calculate the mass concentration of total flavonoids in the sample solution (mg/mL). TFC was expressed in milligrams of rutin per gram of crude extract, i.e., mg RE/g.

### 2.7. Antioxidant activity

The fungal extracts were prepared into a series of sample solutions with a concentration gradient (0.01, 0.05, 01, 0.2, 0.3, 0.4, 0.5, 0.7, 0.9 mg/mL). The antioxidant activity of the crude extract *in vitro* was determined by four experiments, including superoxide anion, DPPH, ABTS and hydroxyl radical scavenging activity (Hou et al., 2020; Wu et al., 2020; Zhang et al., 2020; Nataraj et al., 2022). Ascorbic acid (Vc) was used as a positive control, and the experiment was repeated three times in each group.

#### 2.7.1. 2,2-Diphenyl-1-picrylhydrazyl radical scavenging activity assay

The sample solution of 100 μL was mixed with 100 μL DPPH ethanol solution (0.2 mM), and absorbance was measured at 517 nm after reaction for 30 min at room temperature and away from light.


$$\mathrm{Scavenging}\mathrm{rate}\left(\%\right)=\left[1-\left({\text{A}}_{1}-{\text{A}}_{2}\right)/{\text{A}}_{0}\right]\times 100\%$$

where A_0_ is the absorbance of 100 μL DPPH plus 100 μL ethanol, A_1_ is the absorbance of 100 μL DPPH plus 100 μL sample solution, and A_2_ is the absorbance of 100 μL of sample solution plus 100 μL of ethanol.

#### 2.7.2. Hydroxyl radical scavenging activity assay

The 100 μL sample solution was added to 20 μL salicylic acid solution (6 mM), 20 μL ferrous sulfate solution (6 mM), and 20 μL hydrogen peroxide solution (6 mM). The mixture was thoroughly mixed and reacted at 37°C for 30 min. Absorbance was measured at 510 nm.


$$\mathrm{Scavenging}\mathrm{rate}\left(\%\right)=\left[1-\left({\text{A}}_{1}-{\text{A}}_{2}\right)/{\text{A}}_{0}\right]\times 100\%$$

where A_0_ is the absorbance of 100 μL deionized water plus salicylic acid solution (6 mM), ferrous sulfate solution (6 mM), and hydrogen peroxide (6 mM) each at 20 μL; A_1_ is the absorbance of salicylic acid solution (6 mM), ferrous sulfate solution (6 mM), and hydrogen peroxide (6 mM) each at 20 μL plus 100 μL sample solution; and A_2_ is the absorbance of 100 μL sample solution plus salicylic acid solution (6 mM), ferrous sulfate solution (6 mM), and deionized water (instead of hydrogen peroxide) each at 20 μL.

#### 2.7.3. Superoxide radical scavenging assay

A total of 150 μL of Tris–HCl (0.05 mol/ml, pH 8.2) and 50 μL of sample solution were added to a 96-well plate, mixed and incubated at 25°C for 30 min. Then, 20 μL of freshly prepared 25 mmol/mL ortho-pyrogallol was added, incubated at 25°C for 30 min, and 25 μL of HCl (10 mol/mL) was quickly added to terminate the reaction, and the absorbance was determined at 325 nm.


$$\mathrm{Scavenging}\mathrm{rate}\left(\%\right)=\left[1-\left({\text{A}}_{1}-{\text{A}}_{2}\right)/{\text{A}}_{0}\right]\times 100\%$$

where A_0_ is the absorbance of 50 μL deionized water after mixing the sample solution with the reaction solution, A_1_ is the absorbance of the sample after reaction with the reaction solution, and A_2_ is the absorbance of 20 μL deionized water mixed with sample solution instead of o-nitrophenol.

#### 2.7.4. ABTS radical scavenging activity assay

Accurately weighed ABTS powder (78 mg) and potassium persulfate (13.2 mg) were mixed into 20 mL ultraclean water, preserved at 4°C, stored in dark for 16 h, and used as ABTS reserve solution after stability. Before the experiment, the ABTS reserve solution was diluted with anhydrous ethanol to an absorbance of 0.7 ± 0.02, which was used as the ABTS working solution. The absorbance was measured at 734 nm after 20 μL of sample solution was mixed with 180 μL of ABTS working solution and reacted at room temperature for 6 min away from light.


$$\mathrm{Scavenging}\mathrm{rate}\left(\%\right)=\left[1-\left({\text{A}}_{1}-{\text{A}}_{2}\right)/{\text{A}}_{0}\right]\times 100\%$$

where A_0_ is the absorbance of 180 μL ABTS plus 20 μL deionized water, A_1_ is the absorbance of 180 μL ABTS plus 20 μL sample solution, and A_2_ is the absorbance of 20 μL of sample liquid plus 180 μL of deionized water.

### 2.8. Minimal inhibitory concentration (MIC) and minimum bactericidal concentration (MBC)

The MIC and MBC of crude extracts were evaluated by the 96-well plate method (Wiegand et al., 2008; Li et al., 2016; Zhang Y. et al., 2017; Dydak et al., 2021). Luria-Bertani (LB) liquid medium (100 μL) was added to each well to dilute the crude extract solution, and then 100 μL of 10 mg/mL crude extract solution filtered through a 0.22 μm microporous membrane was added to the first well. The 100 μL mixture from the previous well was transferred to the next well, and the process was repeated to the ninth well. The 100 μL mixture from the ninth (last) well was discarded. The crude extract was diluted to 9 concentrations of 5, 2.5, 1.25, 0.625, 0.312, 0.156, 0.0781, 0.03905, and 0.15475 mg/mL. Then, 20 μL of bacterial solution (OD_620_ = 0.08–0.1, equivalent to 1 × 10^8^ CFU/mL) was successively added to the 96-well plate. LB liquid medium with bacterial suspension and 5% DMSO (LB liquid medium preparation) with bacterial suspension were used as blank controls, and LB liquid medium without bacterial suspension was the negative control. Positive controls were 100 μg/mL streptomycin sulfate prepared with LB liquid medium and 100 μg/mL ampicillin sodium prepared with LB liquid medium. After incubation at 37°C for 24 h, the MIC value was determined by the concentration between clarification and turbidity. The 20 μL clarifying solution was transferred to an LB culture plate and cultured in a 37°C incubator for 24 h. The minimum concentration of crude extract in the medium without bacterial growth was the MBC. All experiments were repeated three times for each type of bacteria.

### 2.9. Detection of bioactive compounds by liquid chromatography-Mass spectrometry (LC-MS) analysis

The LC-MS (Waters, UPLC; Thermo, Q Exactive) analysis platform was used. Compounds were isolated on an ACQUITY UPLC HSS T3 column (2.1 mm 100 mm 1.8 μm). The mobile phase was 0.05% ammonium water (A)/acetonitrile (B) at a flow rate of 0.3 mL min^–1^. The injection volume was 5 μL, and the automatic injector temperature was 4°C. The gradient elution procedure is shown in Supplementary Table 1. For mass spectrometry (MS) detection, ionization was performed in ESI + mode (heater temp, 300°C; sheath gas flow rate, 45 arb; Aux gas flow rate, 15 arb; sweep gas flow rate, 1 arb; spray voltage, 3.0 KV; capillary temperature, 350°C; and S-Lens RF level, 30%.) and ESI- mode (heater temp, 300°C; sheath gas flow rate, 45 arb; aux gas flow rate, 15 arb; sweep gas flow rate, 1 arb; spray voltage, 3.2 KV; capillary temperature, 350°C; and S-Lens RF level, 60%). Full scans (MS1) were performed from 70∼1050 m/z with a resolution of 70,000, and data-dependent two-stage mass spectrometry (DDMS2, TOPN = 10) was performed with a resolution of 17,500. The obtained mass spectra were analyzed with Compound Discoverer 2.0 (Thermo Scientific), and the integrated mzcloud, metlin, and hmdb databases were used to detect secondary metabolites in an untargeted method. The scanning modes were full scan (m/z 70∼1050) and data-dependent secondary mass spectrometry (dd-MS2, TopN = 10). The resolution was 70,000 (primary mass spectrometry) and 17,500 (secondary mass spectrometry). The collision mode was high-energy collision dissociation (HCD). The obtained mass spectra were analyzed using Compound Discoverer (Thermo Scientific), and the secondary metabolites were detected by non-targeted methods using the integrated mzcloud, metlin, and hmdb databases.

### 2.10. Data analysis

All experimental data are expressed as the mean ± SD from three independent observations. The data were analyzed by one-way analysis of variance (ANOVA) followed by Duncan’s multiple range test using SPSS 27.0 software. *P* < 0.05 was used to define statistically significant differences between the control group and experimental group.

## 3. Results

### 3.1. High-throughput sequencing statistics of endophytic fungal communities in AOJ

**FIGURE 2:**
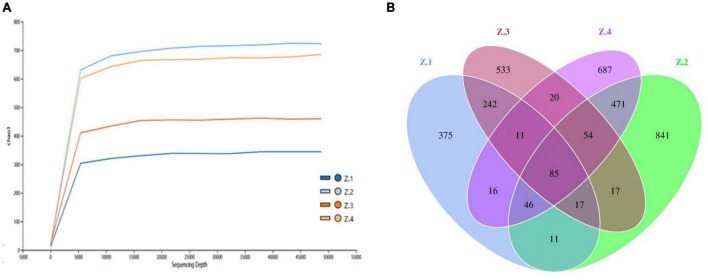
High throughput sequencing. **(A)** ASV rarefaction curves. **(B)** Venn diagram showing the number of shared and specific ASVs for each group.

High-throughput sequencing technology was used to study the diversity of fungi in roots and stems of AOJ with different shapes. A total of 1,221,926 raw PE reads were obtained from 12 samples by high-throughput sequencing. After quality control, the effective tags of fungal sequences in each sample ranged from 51,680 to 112,590, and a total of 3,426 amplicon sequence variants (ASVs) were identified, belonging to 9 phyla, 27 classes, 64 orders, 152 families, and 277 genera. The ASV rarefaction curves are shown in Figure 2A. The curves of the four groups of samples all tended to be flat, indicating that the sequencing data amount was reasonable and could represent the fungal diversity of the samples. As shown in Figure 2B, Z1 shared 355 ASVs with Z3, and Z2 shared 656 ASVs with Z4. The unique ASVs among all samples collected in the Z1, Z2, Z3, and Z4 groups were 375, 533, 687, and 841, respectively. These results indicated that there were significant differences in endophytic fungal communities between Z1 and Z3 and between Z2 and Z4, which might be the cause of the round and triangular shape of the stems of AOJ (Harrison et al., 2021).

### 3.2. Alpha diversity

There were differences in the alpha diversity index of fungal communities in the 12 samples. It can be seen from Table 1 that the coverage index of each sample was close to 1, indicating the integrity of the tested samples; that is, the sequencing result could reflect the real situation of the fungal community composition in the tested samples. The Chao1 index of the Z2 group was the largest, indicating that the sample community contained the largest number of low abundance species. Both the Shannon index and Simpson index of Group Z3 were the highest, indicating that the community diversity and species evenness of Group Z3 were the highest. The lowest Chao1 and Shannon indices were found in the Z1 group, indicating that this group had the fewest species with low abundance and the lowest community diversity. The Simpson index of the Z4 group was the smallest, indicating that the species uniformity of this group was the lowest.

**TABLE 1:** Alpha diversity index statistics.

Sample name	Goods coverage	Chao1	Shannon	Simpson
Z1	1.000 ± 0	368.352 ± 92.289	4.589 ± 0.661	0.860 ± 0.021
Z2	0.999 ± 0	744.586 ± 39.785	5.214 ± 0.260	0.833 ± 0.049
Z3	1.000 ± 0	489.588 ± 85.382	5.618 ± 0.180	0.935 ± 0.015
Z4	0.999 ± 0	661.080 ± 123.761	5.154 ± 0.409	0.812 ± 0.059

Values in the table are mean ± standard deviation.

### 3.3. Beta diversity

**FIGURE 3:**
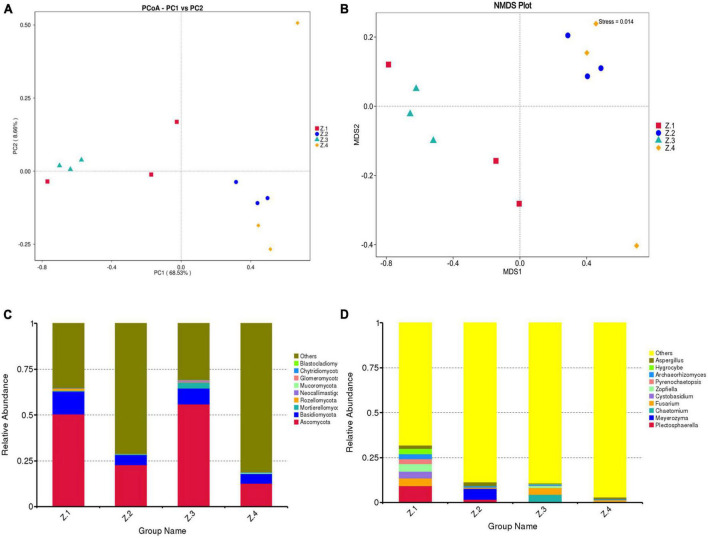
Composition of endophytic fungi in root and stem of AOJ. **(A)** Multiple sample principal coordinate analysis (PCoA) of the ASV level. **(B)** Multiple sample Non-metric multidimensional scaling (NMDS) of the ASV level. **(C)** Relative abundance of phyla in each sample. **(D)** Relative abundance of genus in each sample.

To explore beta diversity, principal coordinates analysis (PCoA) (Figure 3A) and non-metric multidimensional scaling (NMDS) (Figure 3B) were performed for AOJ samples. The sum of PCoA1 and PCoA2 was 77.19%, with Stress <0.2 in NMDS, indicating that the endophytic fungi population structure of sequenced samples had a high diversity, and NMDS could accurately reflect the degree of difference between samples. Groups Z1 and Z2 and Groups Z3 and Z4 were far apart from each other, indicating that there were significant differences in the endophytic fungal communities of the roots and stems of AOJ with two shapes. Figures 3C, D show the relative abundance of endophytes at the phylum and genus levels, respectively. The dominant phylum in the roots and stems of both AOJ types was Ascomycota (mean relative abundance was 35.46%), followed by Basidiomycota (mean relative abundance was 8.04%). The dominant genus in Z1 was *Plectosphaerella* (mean relative abundance was 9.20%). *Fusarium* was the dominant genus in Z4 (mean relative abundance was 1.17%) and a secondary dominant genus in Z1 (mean relative abundance was 4.32%) and Z3 (mean relative abundance was 3.67%). The dominant genus in Z2 was *Meyerozyma* (mean relative abundance was 6.10%). *Chaetomium* was the dominant genus in Z3 (mean relative abundance was 4.01%).

### 3.4. Isolation and identification of endophytic fungi

A total of 31 endophytic fungi were isolated from the roots and stems of AOJ with different shapes, including 5 and 11 strains from the roots and stems of round AOJ and 5 and 10 strains from the roots and stems of triangular AOJ. All strains were initially identified by colony morphology and microscopy (Supplementary Figures 1, 2), and molecular identification was performed by ITS-rDNA sequence analysis. The closest identified matches are listed in Table 2. The nucleotide sequences of 31 endophytic fungi were more than 98% similar to the best matched nucleotide sequences in the nucleotide database.

**TABLE 2:** The homologous strains of endophyte fungi from *Alisma orientale* (Sam.) Juzep.

NO	Genus	Most closely related strain	Ident (%)	Accession.
SG-2	*Trametes* sp.	*Trametes versicolor*	99.33%	MW742530.1
SG-3	*Plectosphaerella* sp.	*Plectosphaerella oligotrophica*	99.22%	MT447499.1
SG-4	*Chaetomium* sp.	*Chaetomium globosum*	99.63%	MG885813.1
SG-5	*Bjerkandera* sp.	*Bjerkandera adusta*	99.66%	MK809422.1
SG-6	*Pseudeurotium* sp.	*Pseudeurotium* sp.	99.05%	MZ380127.1
SJ-1	*Trametes* sp.	*Trametes versicolor*	99.65%	ON319095.1
SJ-2	*Talaromyces* sp.	*Talaromyces purpureogenus.*	99.28%	ON248285.1
SJ-3	*Penicillium* sp.	*Penicillium digitatum*	98.74%	KY558584.1
SJ-4	*Botrytis* sp.	*Botrytis cinerea*	99.8%	KF533037.1
SJ-5	*Gibberella* sp.	*[Gibberella] fujikuroi var. moniliformis*	99.22%	MW405885.1
SJ-6	*Mucor* sp.	*Mucor hiemalis*	98.21%	LC515165.1
SJ-7	*Bjerkandera* sp.	*Bjerkandera fumosa*	98.5%	KX958033.1
SJ-9	*Bjerkandera* sp.	*Bjerkandera* sp.	99.16%	MT085748.1
SJ-10	*Alternaria* sp.	*Alternaria angustiovoidea*	99.45%	OM236751.1
SJ-11	*Alternaria* sp.	*Alternaria compacta*	99.63%	OM237237.1
YG-1	*Clonostachys* sp.	*Clonostachys rosea*	99.63%	MW113577.1
YG-2	*Chaetomium* sp.	*Chaetomium globosum*	99.45%	MT510030.1
YG-4	*Pleosporales* sp.	*uncultured Pleosporales*	98.61%	OM106656.1
YG-5	*Talaromyces* sp.	*Talaromyces marneffei*	98.77%	MF683085.1
YG-6	*Paraphaeosphaeria* sp.	*Paraphaeosphaeria* sp.	99.46%	MT771337.1
YJ-1	*Cladosporium* sp.	*Cladosporium cladosporioides*	99.42%	ON790391.1
YJ-3	*Bjerkandera* sp.	*Bjerkandera fumosa*	98.5%	KX958033.1
YJ-4	*Alternaria* sp.	*Alternaria* sp.	99.26%	MK037447.1
YJ-5	*Trametes* sp.	*Trametes versicolor*	99.48%	ON319098.1
YJ-6	*Botrytis* sp.	*Botrytis cinerea*	99.61%	ON566789.1
YJ-7	*Stagonosporopsis* sp.	*Stagonosporopsis cucurbitacearum*	98.82%	EF107642.1
YJ-8	*Bjerkandera* sp.	*Bjerkandera adusta*	99.66%	OK184566.1
YJ-9	*Alternaria* sp.	*Alternaria* sp.	99.44%	KX064970.1
YJ-10	*Alternaria* sp.	*Alternaria* sp.	99.62%	ON435682.1
YJ-12	*Plectosphaerella* sp.	*Plectosphaerella cucumerina*	99.41%	OM648207.1
YJ-13	*Septoriella* sp.	*Septoriella phragmitis*	99.46%	KT826679.1

**FIGURE 4:**
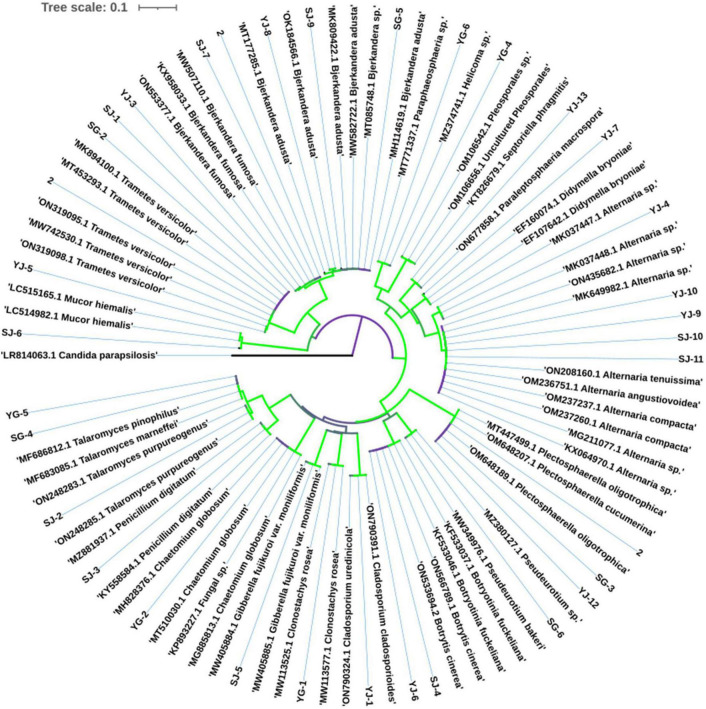
Phylogenetic tree of endophytic fungi in *Alisma orientale* (Sam.) Juzep.

The most common fungi were *Bjerkandera* sp. and *Alternaria* sp., with 5 strains each, and all the endophytic fungi were divided into 17 genera. The phylogenetic tree of endophytic fungi identified from AOJ is shown in Figure 4.

### 3.5. Screening of polyphenol- and flavonoid-producing endophytic fungi

Chromogenic reactions were performed on 31 endophytic fungi isolated from AOJ, and the chromogenic reaction results are shown in Supplementary Figure 3. The results showed that SJ-11, YG-2, YJ-4, YJ-10, YJ-9, and SJ-2 were positive in the color reaction of flavonoids and phenols, indicating that these 6 strains could produce flavonoids and phenolic compounds.

### 3.6. Determination of TPC and TFC

**FIGURE 5:**
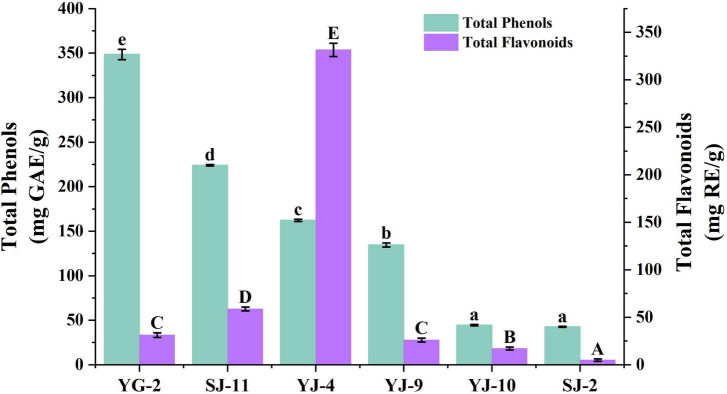
Total Phenol and Total Flavonoid Content of YG-2, SJ-11, YJ-4, YJ-9, YJ-10, and SJ-2 extracts. a–e and A–E: significant differences between different groups (*p* < 0.05).

The Folin–Ciocalteu assay was used to measure the TPC, and the aluminum nitrate colorimetric method was used to measure the TFC. The experimental results are shown in Table 3 and Figure 5. The TPC of the fungal YG-2 extract was the highest, 348.67 ± 5.90 mg GAE/g. In addition, the extracts of fungi SJ-11, YJ-4, and YJ-9 showed higher TPC. The TPC and TFC in the extracts of SJ-2 were the lowest, 42.75 ± 0.48 mg GAE/g and 5.12 ± 1.26 mg RE/g, respectively. The JY-4 extract had the highest TFC (331.66 ± 6.93 mg RE/g).

**TABLE 3:** Total phenol and total flavonoid content.

Crude extract	TPC(mg GAE/g)	TFC(mg RE/g)
YG-2	348.67 ± 5.90^e^	31.33 ± 2.46^C^
SJ-11	224.21 ± 0.79^d^	58.84 ± 1.99^D^
YJ-4	162.30 ± 1.32^c^	331.66 ± 6.93^E^
YJ-9	134.77 ± 2.30^b^	26.02 ± 2.08^C^
YJ-10	44.57 ± 0.90^a^	17.23 ± 1.51^B^
SJ-2	42.75 ± 0.48^a^	5.12 ± 1.26^A^

^a−e^ and ^A−E^ Significant differences between different groups (*p* < 0.05).

### 3.7. Antioxidant activity

**FIGURE 6:**
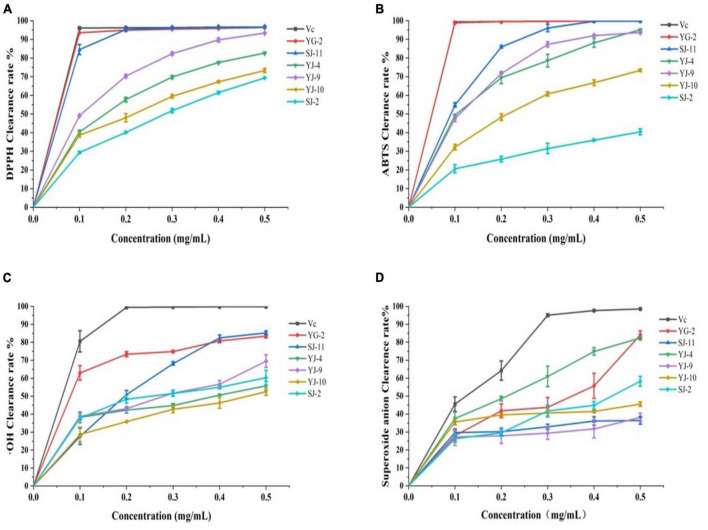
Antioxidant activity *in vitro* assays of YG-2, SJ-11, YJ-4, YJ-9, YJ-10, and SJ-2 extracts. **(A)** DPPH clearance assay; **(B)** ABTS clearance assay; **(C)** ⋅OH clearance assay; **(D)** superoxide anion clearance assay.

DPPH, ABTS, hydroxyl (⋅OH) radical and superoxide anion (⋅O_2_^–^) scavenging activities were used to analyze the *in vitro* antioxidant activities of endophytic fungi crude extracts. The experimental results are shown in Table 4 and Figure 6. As shown in Figure 6, all samples showed concentration-dependent scavenging activity against all free radicals within the experimental concentration range. In DPPH, ABTS and hydroxyl radical scavenging tests, the scavenging ability of YG-2, SJ-11, YJ-4, YJ-9, YJ-10, and SJ-2 decreased gradually. Except for SJ-2, the scavenging activities of other strains on DPPH and ABTS free radicals were stronger than those on ⋅OH and ⋅O_2_^–^. The crude extract of YG-2 showed the strongest free radical scavenging activity, with an IC_50_ *_*DPPH*_* of 0.009 ± 0.000 mg/mL, IC_50_ *_*ABTS*_* of 0.023 ± 0.002 mg/mL and IC_50_ *_⋅*OH*_* of 0.081 ± 0.006 mg/mL. Significantly higher than that of other crude extracts (Table 4, *P* < 0.05). At the maximum concentration of 0.5 mg/mL, the scavenging activity of YG-2 on DPPH and ABTS was the highest (96.39 ± 0.20% and 99.82 ± 0.07%, respectively), which was close to that of the control group (96.62 ± 0.28% and 99.85 ± 0.06%, respectively). The scavenging activities of YG-2 and SJ-11 were 83.44 ± 1.05% and 85.24 ± 0.24% at 0.5 mg/mL, respectively, compared with other crude extracts. The crude extract of YJ-4 had the strongest superoxide anion scavenging activity, and its IC_50_*_⋅*O2*–_* was 0.176 ± 0.01 mg/mL. The superoxide anion scavenging rate was 82.37 ± 0.99% at 0.5 mg/mL. In conclusion, the YG-2 crude extract had the strongest free radical scavenging ability, while SJ-2 had the weakest free radical scavenging ability.

**TABLE 4:** IC_50_ value from antioxidant activity *in vitro* assays of YG-2, SJ-11, YJ-4, YJ-9, YJ-10, and SJ-2 extracts.

Crude extract	IC_50_(mg/mL)			
	DPPH	ABTS	Hydroxyl radical	Superoxide anion clearance assay
Vc	0.009 ± 0.001^a^	0.021 ± 0.001^a^	0.042 ± 0.003^a^	0.119 ± 0.007^a^
YG-2	0.009 ± 0.000^a^	0.023 ± 0.002^a^	0.081 ± 0.006^a^	0.260 ± 0.030^c^
SJ-11	0.029 ± 0.001^b^	0.063 ± 0.006^b^	0.153 ± 0.015^b^	nd
YJ-4	0.144 ± 0.001^d^	0.109 ± 0.003^c^	0.385 ± 0.057^d^	0.176 ± 0.017^b^
YJ-9	0.107 ± 0.001^c^	0.109 ± 0.003^c^	0.236 ± 0.016^c^	nd
YJ-10	0.186 ± 0.003^e^	0.204 ± 0.008^d^	0.479 ± 0.055^e^	nd
SJ-2	0.258 ± 0.005^f^	nd	0.252 ± 0.028^c^	0.437 ± 0.013^d^

^a−f^ Significant differences between different groups (*p* < 0.05). nd, not detected (the result higher 0.5 mg/mL).

### 3.8. Antibacterial activity

The antibacterial activity of the crude extract was evaluated by measuring the MIC and MBC values, and the results are shown in Table 5 and Supplementary Figure 4. The crude extracts from the fermentation broth of 6 strains of fungi had antibacterial effects on *Escherichia coli*, *Bacillus subtilis*, *Pseudomonas aeruginosa*, and *Staphylococcus aureus*. Among them, YG-2 had the best inhibitory effect against *S. aureus*, *B. subtilis*, *P. aeruginosa*, and *E. coli*, with MIC values of 0.625, 0.625, 1.25, and 1.25 mg/mL, respectively, indicating that YG-2 had a better inhibitory effect against gram-positive bacteria than gram-negative bacteria. However, from the point of view of minimum bactericidal concentration, SJ-11 has the best bactericidal effect, and its MBC value against *E. coli* is 2.5 mg/mL. In general, the antibacterial effect was YG-2 > SJ-11 > YJ-4 > YJ-10 > YJ-9, and SJ-2.

**TABLE 5:** Determination of MIC (mg/mL) and MBC (mg/mL) of YG-2, SJ-11, YJ-4, YJ-9, YJ-10 and SJ-2 extracts.

Crude extract	Gram-positive bacteria				Gram-negative bacteria			
	*B. subtilis*		*S. aureus*		*P. aeruginosa*		*E. coli*	
	MIC	MBC	MIC	MBC	MIC	MBC	MIC	MBC
YG-2	0.625	5	0.625	5	1.25	5	1.25	5
SJ-11	2.5	5	2.5	5	2.5	5	1.25	2.5
YJ-4	2.5	5	2.5	nd	2.5	nd	1.25	nd
YJ-9	5	nd	5	nd	5	nd	5	nd
YJ-10	2.5	5	2.5	5	2.5	5	2.5	5
SJ-2	5	nd	5	nd	5	nd	5	nd

nd, not detected (the result higher 5 mg/mL).

### 3.9. Liquid chromatography-Mass spectrometry

The crude extracts of fungi YG-2, SJ-11, YJ-4, and YJ-9 had high contents of total phenols and total flavonoids and had good antioxidant and antibacterial activities. LC-MS was further used to analyze the chemical constituents. The results are shown in Table 6, chromatograms of the four samples are shown in Supplementary Figure 5, and mass spectra of the main components of the four samples are shown in Supplementary Figure 6. The identified phenolic substances include non-flavonoid phenols (altenuene, phloroglucinol), flavonoids (glycitein, daidzein), phenolic acids (ferulic acid, caffeic acid), and flavonolignan (silibinin). A total of 55 compounds were identified in the YG-2 crude extract, of which caffeic acid was the main compound with a concentration of 10.12 μmol/g. The main compounds identified from the crude extracts of SJ-11 and YJ-9 were Skatole with concentrations of 5.53 μmol/g and 2.62 μmol/g, and the total compounds were 52 and 50, respectively. A total of 54 compounds were identified in the crude extract of YJ-4, of which adenine was the main compound with a concentration of 7.11 μmol/g. The results showed that the extracts of the four strains of fungi were rich in secondary metabolites with biological activity, and phenols and flavonoids were detected in all samples.

**TABLE 6:** The identification of the chemical composition of endophytic fungi extracts by LC-MS analysis.

NO	Classification	Name of identified compound	RT (min)	Formula	m/z	Adduction	Endophytic fungi (μmol/g)			
							YG-2	SJ-11	YJ-4	YJ-9
1	Flavonoid	Silibinin	5.995	C_25_ H_22_ O_10_	483.128	[M + H]	0.01	nd	0.03	0.12
2	Phenol	Altenuene	5.234	C_15_ H_16_ O_6_	293.101	[M + H]	0.01	0.07	0.01	nd
3	Flavonoid	Glycitein	4.429	C_16_ H_12_ O_5_	285.075	[M + H]	0.31	0.01	0.10	0.03
4	Flavonoid	Daidzein	4.617	C_15_ H_10_ O_4_	255.065	[M + H]	0.07	0.20	0.13	0.12
5	Phenol	Ferulic acid	4.336	C_10_ H_10_ O_4_	195.065	[M + H]	0.04	0.06	0.59	0.12
6	Phenol	Caffeic acid	4.532	C_9_ H_8_ O_4_	181.049	[M + H]	10.12	0.03	0.02	0.01
7	Phenol	Phloroglucinol	4.102	C_6_ H_6_ O_3_	127.039	[M + H]	0.08	0.14	0.06	0.06
8	Non-phenolic	Skatole	4.261	C_9_ H_9_ N	132.080	[M + H]	0.05	5.36	0.06	2.62
9	Non-phenolic	Adenine	0.883	C_5_ H_5_ N_5_	136.062	[M + H]	0.16	0.75	7.11	0.07
10	Non-phenolic	Cinnamic acid	4.563	C_9_ H_8_ O_2_	149.060	[M + H]	0.03	0.15	1.32	0.14
11	Non-phenolic	LYSINE	8.904	C_6_ H_14_ N_2_ O_2_	331.180	[2M + K]	0.61	0.00	0.01	nd
12	Non-phenolic	4-Methyl-5-thiazoleethanol	2.583	C_6_ H_9_ N O S	144.048	[M + H]	0.11	0.84	0.04	0.90
13	Non-phenolic	Kojic acid	3.362	C_6_ H_6_ O_4_	143.034	[M + H]	0.44	0.09	1.46	0.16
14	Non-phenolic	Veratrole	6.904	C_8_ H_10_ O_2_	121.065	[M + H-H_2_O]	0.57	0.04	0.53	0.06
15	Non-phenolic	Hypoxanthine	1.236	C_5_ H_4_ N_4_ O	137.046	[M + H]	0.00	0.04	1.06	0.03
16	Non-phenolic	5-Hydroxyindole	1.301	C_8_ H_7_ N O	134.060	[M + H]	0.13	0.61	0.81	1.70
17	Non-phenolic	L-Norleucine	6.852	C_6_ H_13_ N O_2_	132.102	[M + H]	2.49	nd	nd	nd
18	Non-phenolic	Thymine	1.719	C_5_ H_6_ N_2_ O_2_	127.050	[M + H]	0.59	nd	0.20	0.01
19	Non-phenolic	4-Hydroxy-6-methyl-2-pyrone	3.54	C_6_ H_6_ O_3_	127.039	[M+H]	0.03	0.31	4.77	0.64
20	Non-phenolic	Pyrrole-2-carboxylic acid	2.199	C_5_ H_5_ N O_2_	112.039	[M + H]	0.03	0.51	0.05	0.72
21	Non-phenolic	Protoporphyrin IX	8.229	C_34_ H_34_ N_4_ O_4_	563.274	[M + H]	0.31	nd	nd	nd
22	Non-phenolic	Carviolin	4.558	C_16_ H_12_ O_6_	301.070	[M + H]	0.19	0.02	1.04	0.01
23	Non-phenolic	Sphingosine	8.261	C_18_ H_37_ N O_2_	300.289	[M + H]	nd	0.05	0.02	0.51
24	Non-phenolic	Oleamide	7.897	C_18_ H_35_ N O	282.279	[M + H]	nd	0.00	0.85	nd
25	Non-phenolic	Pyridoxine	1.231	C_8_ H_11_ N O_3_	170.081	[M + H]	0.01	0.15	0.04	0.19
26	Non-phenolic	4-Phenylbutyric acid	5.336	C_10_ H_12_ O_2_	165.091	[M + H]	0.15	0.05	0.75	0.08
27	Non-phenolic	Arecoline	3.525	C_8_ H_13_ N O_2_	156.102	[M + H]	0.03	0.20	0.10	0.25
28	Non-phenolic	Citral	8.154	C_10_ H_16_ O	135.117	[M + H-H_2_O]	0.12	0.40	0.23	0.13
29	Non-phenolic	4-Anisic acid	5.233	C_8_ H_8_ O_3_	153.054	[M + H]	0.29	0.07	0.05	0.08
30	Non-phenolic	3-Methoxyindole	2.758	C_9_ H_9_ N O	148.075	[M + H]	0.11	0.25	0.40	0.12
31	Non-phenolic	Gentisyl alcohol	2.731	C_7_ H_8_ O_3_	141.054	[M + H]	0.03	0.02	0.18	0.17
32	Non-phenolic	Nicotinic acid	0.99	C_6_ H_5_ N O_2_	124.039	[M + H]	0.32	0.03	0.20	0.05
33	Non-phenolic	Isobutyric acid	11.657	C_4_ H_8_ O_2_	177.112	[2M + H]	0.02	0.21	0.13	0.34
34	Non-phenolic	Piperidine	4.577	C_5_ H_11_ N	86.097	[M + H]	0.08	0.02	0.75	0.02
35	Non-phenolic	Erucamide	12.868	C_22_ H_43_ N O	338.340	[M + H]	0.01	0.03	0.17	0.08
36	Non-phenolic	Evernic acid	4.614	C_17_ H_16_ O_7_	333.096	[M + H]	0.11	0.03	0.05	0.05
37	Non-phenolic	Phytosphingosine	7.439	C_18_ H_39_ N O_3_	318.300	[M + H]	0.06	0.05	0.04	0.04
38	Non-phenolic	Glutathione	7.575	C_10_ H_17_ N_3_ O_6_ S	308.091	[M + H]	0.15	nd	nd	nd
39	Non-phenolic	Kinetin	3.98	C_10_ H_9_ N_5_ O	216.088	[M + H]	0.02	0.04	0.13	0.03
40	Non-phenolic	L-Dopa	11.308	C_9_ H_11_ N O_4_	198.076	[M + H]	0.10	0.01	nd	nd
41	Non-phenolic	LL-2,6-Diaminoheptanedioate	5.957	C_7_ H_14_ N_2_ O_4_	191.106	[M + H]	0.02	0.11	0.70	0.03
42	Non-phenolic	4-Methoxycinnamic acid	8.818	C_10_ H_10_ O_3_	179.070	[M + H]	0.03	0.01	0.40	0.08
43	Non-phenolic	6-Pentyl-2H-pyran-2-one	7.555	C_10_ H_14_ O_2_	149.096	[M + H-H_2_O]	0.14	0.14	0.06	0.16
44	Non-phenolic	Apocynin	6.908	C_9_ H_10_ O_3_	167.070	[M + H]	0.23	0.04	0.68	0.04
45	Non-phenolic	L-Phenylalanine	3.105	C_9_ H_11_ N O_2_	166.086	[M + H]	0.01	0.10	0.07	0.06
46	Non-phenolic	4-Coumaric acid	6.257	C_9_ H_8_ O_3_	165.055	[M + H]	0.19	0.02	0.17	0.11
47	Non-phenolic	N-Methylanthranilic Acid	7.028	C_8_ H_9_ N O_2_	152.070	[M + H]	nd	0.04	0.35	0.03
48	Non-phenolic	Thymol	5.068	C_10_ H_14_ O	151.112	[M + H]	0.09	0.06	0.02	0.02
49	Non-phenolic	4-Hydroxybenzoic acid	6.011	C_7_ H_6_ O_3_	139.039	[M + H]	0.02	0.02	0.26	0.06
50	Non-phenolic	3,4-Dihydroxybenzaldehyde	4.663	C_7_ H_6_ O_3_	139.039	[M + H]	0.14	0.04	0.14	0.10
51	Non-phenolic	Trigonelline	7.556	C_7_ H_7_ N O_2_	138.055	[M + H]	0.00	0.04	0.01	0.11
52	Non-phenolic	4-Hydroxyindole	3.895	C_8_ H_7_ N O	134.060	[M + H]	0.03	0.06	0.48	0.09
53	Non-phenolic	L-Pyroglutamic acid	1.249	C_5_ H_7_ N O_3_	130.050	[M + H]	0.06	nd	0.06	nd
54	Non-phenolic	Maltol	3.169	C_6_ H_6_ O_3_	127.039	[M + H]	0.02	0.09	0.10	0.02
55	Non-phenolic	Uracil	1.261	C_4_ H_4_ N_2_ O_2_	113.035	[M + H]	0.01	0.01	0.15	0.05
56	Non-phenolic	Histamine	14.542	C_5_ H_9_ N_3_	112.087	[M + H]	0.09	0.11	0.11	0.09
57	Non-phenolic	Choline	0.758	C_5_ H_13_ N O	104.107	[M + H]	0.07	0.05	0.09	0.02
58	Non-phenolic	Aniline	1.231	C_6_ H_7_ N	94.066	[M + H]	0.03	0.06	0.02	0.09

nd, not detect.

## 4. Discussion

With the improvement of people’s living standards, the need for natural antioxidants and antibacterial drugs has become very important. The use of endophytic fungi to produce natural antioxidants and antibacterial drugs has been widely studied. It is of great significance to carry out scientific research on AOJ endophytic fungi to increase the yield potential of Chinese medicinal materials and realize the healthy cultivation of AOJ. In this study, the diversity and community structure of AOJ endophytic fungi were preliminarily described, and endophytic fungi with high phenolic production were screened. The results showed that the endophytic fungi of AOJ had strong antioxidant activity and antibacterial effects on four kinds of pathogenic bacteria, suggesting that AOJ endophytic fungi have potential as sources of antioxidants and antibacterial drugs.

Endophytic fungi have mutualism with host plants, which is an important factor affecting plant productivity and health. The composition and structure of endophytic fungi are different in different plant tissues, and some endophytic fungi only exist in specific plant tissue parts. The tissue specificity of endophytic fungi is an important factor for the accumulation of bioactive substances in different tissues (Park et al., 2012; Fan S. et al., 2020). The diversity and community composition of endophytic fungi can provide new insights for the study of AOJ and endophytic fungi.

In this study, stems and roots were collected from two kinds of AOJ, and a high-throughput sequencing method overcame the problem that traditional culture could not isolate all endophytic fungi and effectively revealed the diversity and community structure of endophytic fungi in different parts of AOJ with different shapes. The Venn diagram initially revealed the ASV differences of endophytic fungi in different parts of AOJ with the same shape and in the same parts of AOJ with different shapes. There were 803, 1542, 979, and 1390 ASVs in the Z1, Z2, Z3, and Z4 groups, respectively, indicating that the number of endophytic fungi in AOJ stems was greater than that in roots, which was consistent with the number of AOJ endophytic fungi isolated from traditional culture. Overall, there were significantly fewer ASVs unique to circular AOJs (908) than triangular AOJs (1519). Although the same ASVs existed in the same part of AOJ with different shapes, there were only 355 ASVs identical to Z1 and Z3 and 375 and 687 ASVs unique to Z1 and Z3, respectively. Similarly, Z2 and Z4 have 656 identical ASVs, while Z2 and Z4 have 533 and 841 unique ASVs, respectively.

Alpha diversity analysis showed that there were significant differences in the community diversity and species evenness of endophytic fungi in different groups. In the root tissue, the Shannon index and Simpson index of the circular AOJ were lower than those of the triangular AOJ, indicating that the diversity and species evenness of the endophytic fungal community in the roots of the circular AOJ were lower than those of the triangular AOJ. The results were reversed in stem tissue, and the Shannon index and Simpson index of the circular AOJ were higher than those of the triangular AOJ, indicating that the diversity and species evenness of the endophytic fungal community in the circular AOJ were higher than those in the triangular AOJ. For AOJs of both shapes, the Chao1 index of roots was lower than that of stems, indicating that there were more low-abundance species in roots than in stems in both AOJs.

In addition, the PCoA and NMDS maps in the results of beta diversity analysis indicated that the fungal communities in root and stem tissues of the two AOJs were different and divided into different communities. The results of diversity analysis showed that the dominant genus of phylum was *Ascomycota* in all the samples, and at the genus level, the dominant genus of phylum was inconsistent in all four groups. Through traditional cultivation methods, 31 endophytic fungi were isolated from AOJ, which were divided into 17 genera. *Bjerkandera* sp. and *Alternaria* sp. were the most common endophytic fungi. This result was different from the result of high-throughput sequencing, which was speculated to be because the number of isolated strains was not sufficient. To obtain more representative data on endophytic fungal diversity, multiple isolates should be used to accumulate a sufficiently large number of endophytic fungi. The reason why AOJs determine whether their stems grow round or triangular during growth is unclear. However, studies have shown that dominant endophytic fungi can have huge ecological consequences, and some studies have shown that endophytic fungi can affect the expression of host genes and thus affect plants (Harrison et al., 2021). In addition, the results of Liu et al. (2022) showed that the endophytic fungus *Epichloë bromicola* can affect the growth of host plants and has a significant effect on the plant height of *Hordeum vulgare*. Therefore, it can be hypothesized that the formation of round AOJs and triangular AOJs may be due to the existence of some endophytic fungi that affect the gene expression or growth of AOJs, and these endophytes are different in AOJ plants with two traits, which are reflected in the significant difference in ASVs and the difference in dominant genera.

The six strains of fungi identified by color reaction belonged to *Talaromyces* sp. (SJ-2), *Alternaria* sp. (SJ-10, SJ-11, YJ-4, YJ-9), and *Chaetomium* sp. (YG-2). *Talaromyces purpureogenus* was isolated from brown algae by Kumari et al. (2018) showed anticancer cell proliferation and antioxidant activity. Kaul et al. (2008) revealed by GC-MS that an *Alternaria* sp. from rose is capable of producing methyl eugenol, which constitutes 1.9% of rose essential oil and is an important bioactive compound. Studies have reported that *Chaetomium* sp. is one of the most bioactive endophytic fungi, and the various metabolites involved in its production have anticancer, antibacterial, antioxidant and other properties (Fatima et al., 2016). Our results showed that the crude extracts of these six strains all contained high TPC and TFC, and the TFC of YJ-4 was higher than that of TPC. The reason why the total flavonoid content was higher than the total phenolic content was the limitation of the measurement method. To date, the Folin–Ciocalteu method is commonly used to determine the content of total phenols (Mekinić et al., 2019), and the aluminum nitrate colorimetric method and aluminum trichloride colorimetric method are commonly used to determine total flavonoids. Even if these methods are generally accepted and used, the presence of other substances in the sample may interfere with experimental results. This question has not been well addressed and has been encountered by other researchers. For example, Li et al. (2022) determined the contents of total phenol and total flavonoid in the crude extracts of endophytic fungi AP-11 and AP-12 of *Andrographis paniculata* (Burm. f.) Nees. The same methods we used, the Folin–Ciocalteu method and aluminum nitrate colorimetric method, were used, and the results showed that the TFC of the two crude extracts was greater than the TPC. The crude extracts of the 6 strains all showed good antioxidant activity, especially YG-2, which had strong antioxidant activity *in vitro*. There was no significant difference between the crude extracts and the positive control ascorbic acid in the scavenging ability of some free radicals. To better understand the antioxidant activity of crude extracts of endophytic fungi such as YG-2, further *in vivo* antioxidant studies should be conducted.

In addition, the crude extracts of the 6 strains showed inhibitory effects on all four kinds of pathogens. MIC values were 0.625-0.5 mg/mL, and MBC values were 2.5 or 5 mg/mL. The crude extract of fungus YG-2 had the highest TPC and the best antibacterial effect, which was consistent with the results of Ju et al. (2018), whose experimental results showed that water extraction of bayberry extract had the highest TPC and antibacterial activity among four solvents. That is, the minimum MIC and MBC of the YG-2 crude extract may be due to its highest TPC. According to our results, MIC and MBC were related to the value of TPC, but the specific relationship needs to be further studied. Moreover, the main antibacterial components of different extracts were different. The antibacterial effect of extracts is not only derived from one compound but may be synergistic by many compounds (Ju et al., 2018). Our LC-MS results showed that the four crude extracts contained more than 6 phenolic compounds, including flavonoids and phenolic acids. Studies have reported that metabolites such as phenolic acids and flavonoids have antibacterial properties (Adamczak et al., 2020). Oliveira et al. (2015) showed that silibinin had a significant inhibitory effect on *E. coli*, and its MIC was 64 μg/mL. Salaheen et al. (2017) studied the antibacterial activity of phenolic acids against methicillin-resistant *Staphylococcus aureus* (MRSA), and the results showed that five phenolic acids, including caffeic acid, completely inhibited vegetative MRSA growth *in vitro*. The four crude extracts not only contain phenols such as silibinin and caffeic acid but also contain organic acids such as cinnamic acid and kojic acid. Adamczak et al. (2020) studied 6 kinds of organic acids from plants. The results showed that all 6 kinds of organic acids have antibacterial activity. In summary, the antibacterial activity of crude extracts may be closely related to phenols and organic acids, and the value of TPC was related to MIC and MBC. Our experimental results preliminarily indicated that AOJ endophytic fungal extracts have antibacterial activity, but the specific antibacterial components are still unclear and need further identification and research.

The crude extracts of four endophytic fungi with strong antioxidant and antibacterial activities were selected for preliminary chemical composition identification by LC-MS. The results of LC-MS showed that the main component of the crude extract of YG-2 was caffeic acid, which is a phenolic acid with antibacterial and antioxidant activities. The results of studies by Spagnol et al. (2019) showed that the IC_50_ value of caffeic acid clearing DPPH was 2.39 μg/mL and that of ABTS was 1.96 μg/mL, both of which were lower than that of ascorbic acid. That is, caffeic acid has better DPPH radical and ABTS radical trapping ability than ascorbic acid, showing excellent antioxidant activity (Spagnol et al., 2019). Our results showed that the IC50 value of the YG-2 crude extract in clearing DPPH and ABTS was slightly greater than or equal to that of ascorbic acid, but there was no significant difference (*p* < 0.05). Therefore, we speculate that caffeic acid is responsible for a significant portion of the antioxidant activity of YG-2, but not all of it.

In addition, the LC-MS method is suitable for the detection of substances with high polarity and has its own limitations. Although our results are significant for the preliminary identification of chemical components in crude extracts, other methods should be added in subsequent studies to compensate for the deficiencies of a single method to better analyze and identify chemical components in crude extracts. In addition, to develop AOJ endophytic fungi for food and medical applications, especially as sources of antioxidants and antimicrobial drugs, further research is needed in the future.

## 5. Conclusion

In summary, this study preliminarily elaborated the diversity of endophytic fungi of AOJ, and the diversity of endophytic fungi in roots and stems of AOJ with different shapes was different. Six strains of YG-2, SJ-11, YJ-4, YJ-9, YJ-10, and SJ-2 fungi with high yields of flavonoids and phenols were isolated from AOJ. The crude extracts of endophytic fungi fermentation broth showed good antioxidant and antibacterial activities, among which the YG-2 crude extract showed the strongest antioxidant and antibacterial activity, and caffeic acid was the main component of the YG-2 crude extract. The results showed that the endophytic fungus YG-2 (*Chaetomium globosum*) had the potential to produce antioxidants.

## Data availability statement

The original contributions presented in the study are included in the article/Supplementary material, further inquiries can be directed to the corresponding author.

## Author contributions

NS, ZC, and GC performed the experiments and authored or reviewed drafts of the manuscript. YQ and WL performed the experiments. YX authored or reviewed drafts of the manuscript. ZT and HC conceived and designed the experiments and approved the final draft. QL and MY analyzed the data. TB contributed reagents, materials, and analysis tools. All authors contributed to the article and approved the submitted version.
