# Supplementary material for: Diversity, chemical constituents and biological activities of endophytic fungi from Alisma orientale (Sam.) Juzep
Source: Front Microbiol. 2023 Jun 21;14:1190624. doi: 10.3389/fmicb.2023.1190624 (PMC10320293; doi:10.3389/fmicb.2023.1190624)
Supplement: Supplementary file 1 [file Table_1.DOCX]

Supplementary Material

Diversity, chemical constituents and biological activities of Endophytic fungi from *Alisma orientale* (Sam.) Juzep.

**Nayu Shen^1^**†**, Zhao Chen^2^**†**, GuiXin Cheng^1^**†**, Wenjie Lin^1^, Yihan Qin^1^, Yirong Xiao^3^, Hui Chen^1^, Zizhong Tang^1*^, Qingfeng Li^1^, Ming Yuan^1^, Tongliang Bu^1^**

*** Correspondence:** Zizhong Tang**:** [14126@sicau.edu.cn](mailto:14126@sicau.edu.cn(Z.Tang))

Table S1 Mobile phase elution procedure

| Time (min) | Flow rate (mL/min) | A (%) | B (%) |
| --- | --- | --- | --- |
| 0.00 | 0.30 | 95 | 5 |
| 1.00 | 0.30 | 95 | 5 |
| 12.50 | 0.30 | 5 | 95 |
| 13.50 | 0.30 | 5 | 95 |
| 13.60 | 0.30 | 95 | 5 |
| 16.00 | 0.30 | 95 | 5 |

Mobile phase A: water (0.05% Ammonium); Mobile phase B: acetonitrile.
