# Supplementary material for: Diversity, chemical constituents and biological activities of endophytic fungi from Alisma orientale (Sam.) Juzep
Source: Front Microbiol. 2023 Jun 21;14:1190624. doi: 10.3389/fmicb.2023.1190624 (PMC10320293; doi:10.3389/fmicb.2023.1190624)
Supplement: Supplementary file 3 [file Image_2.PDF]

Supplementary Material

Diversity, chemical constituents and biological activities of Endophytic fungi from *Alisma orientale* (Sam.) Juzep.

Nayu Shen[1]†, Zhao Chen[2]†, GuiXin Cheng[1]†, Wenjie Lin[1], Yihan Qin[1], Yirong Xiao[3], Hui Chen[1], Zizhong Tang[1*], Qingfeng Li[1], Ming Yuan[1], Tongliang Bu[1]

*** Correspondence:** Zizhong Tang**:** 14126@sicau.edu.cn

SG-2

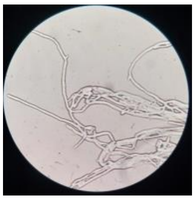

SG-3

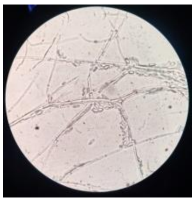

SG-4

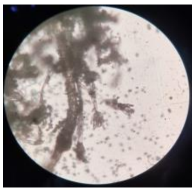

SG-5

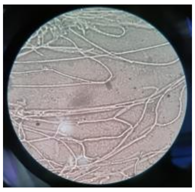

SG-6

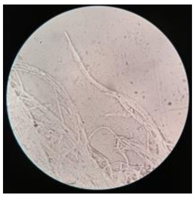

SJ-1

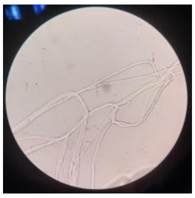

SJ-2

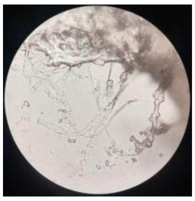

SJ-3

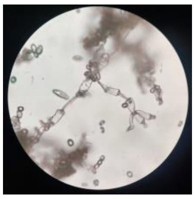

SJ-4

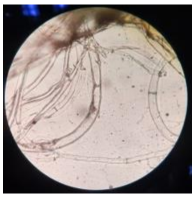

SJ-5

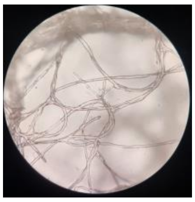

SJ-6

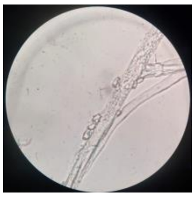

SJ-7

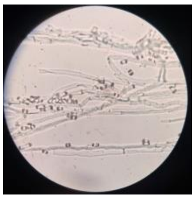

SJ-9

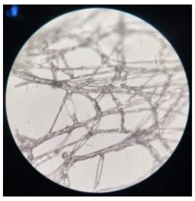

SJ-10

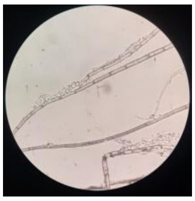

SJ-11

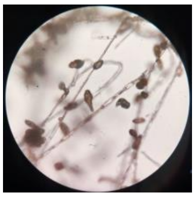

YG-1

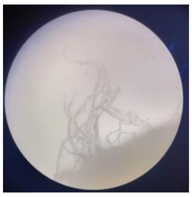

YG-2

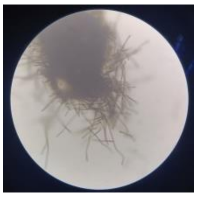

YG-4

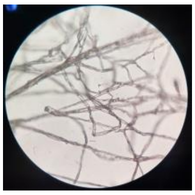

YG-5

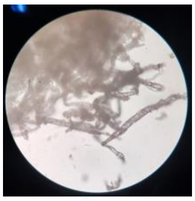

YG-6

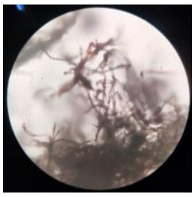

YJ-1

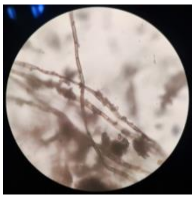

YJ-3

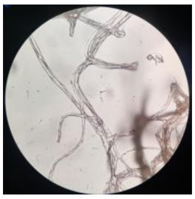

YJ-4

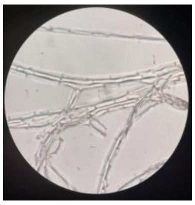

YJ-5

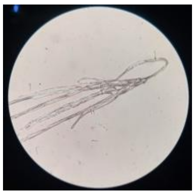

YJ-6

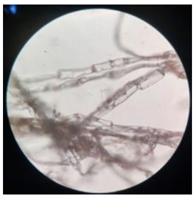

YJ-7 YJ-8 YJ-9 YJ-10 YJ-12

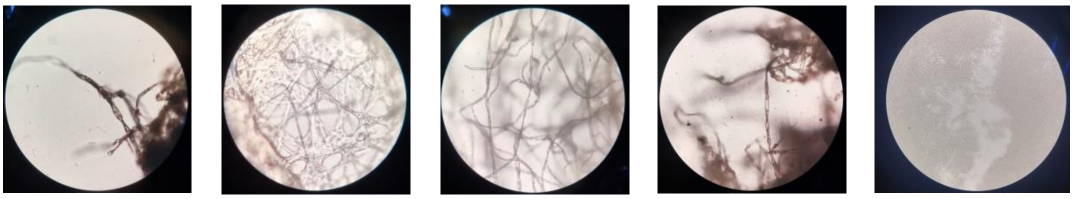

YJ-13

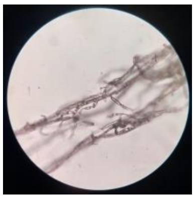

Supplementary Figure 2 The characteristics of mycelium on microscopic view.
