# Supplementary material for: Diversity, chemical constituents and biological activities of endophytic fungi from Alisma orientale (Sam.) Juzep
Source: Front Microbiol. 2023 Jun 21;14:1190624. doi: 10.3389/fmicb.2023.1190624 (PMC10320293; doi:10.3389/fmicb.2023.1190624)
Supplement: Supplementary file 4 [file Image_3.PDF]

Supplementary Material

Diversity, chemical constituents and biological activities of Endophytic fungi from *Alisma orientale* (Sam.) Juzep.

Nayu Shen[1]†, Zhao Chen[2]†, GuiXin Cheng[1]†, Wenjie Lin[1], Yihan Qin[1], Yirong Xiao[3], Hui Chen[1], Zizhong Tang[1*], Qingfeng Li[1], Ming Yuan[1], Tongliang Bu[1]

*** Correspondence:** Zizhong Tang**:** 14126@sicau.edu.cn

	A B C D		A B C D
SJ-1	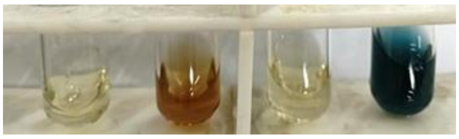	SJ-2	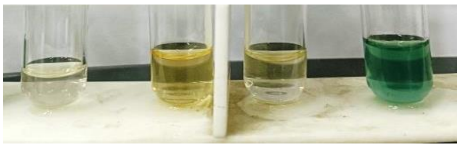
SJ-3	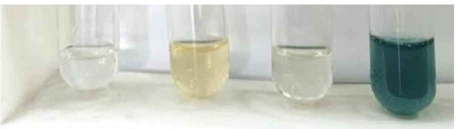	SJ-4	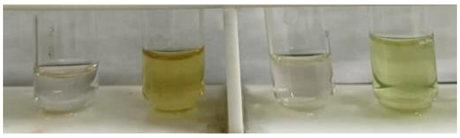
SJ-5	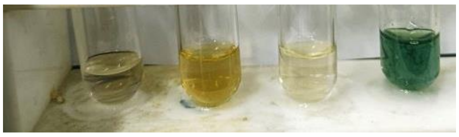	SJ-6	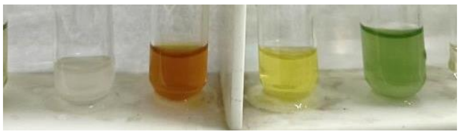
SJ-7	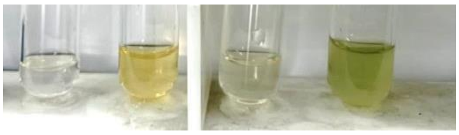	SJ-9	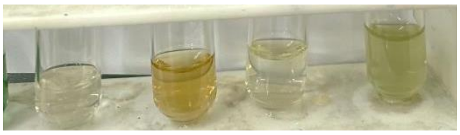
SJ-10	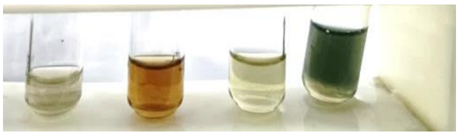	SJ-11	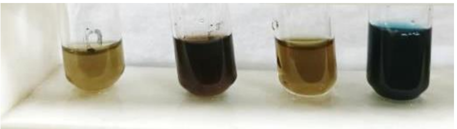
SG-2	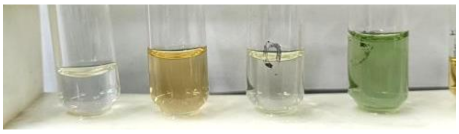	SG-3	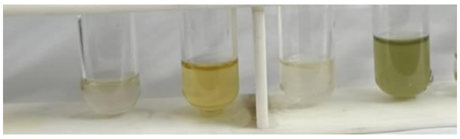
SG-4	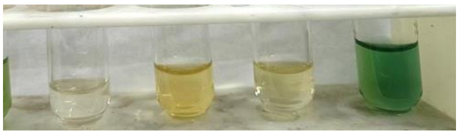	SG-5	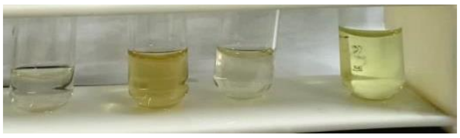
SG-6	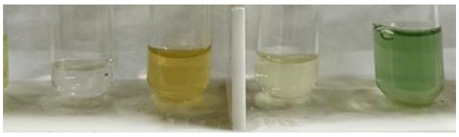	YJ-1	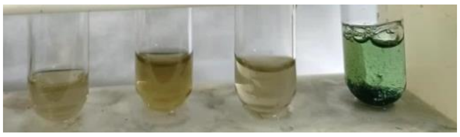
YJ-3	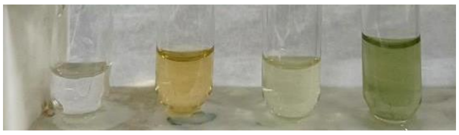	YJ-4	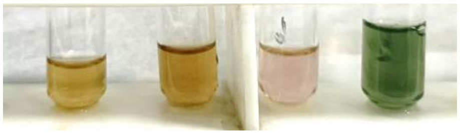
YJ-5	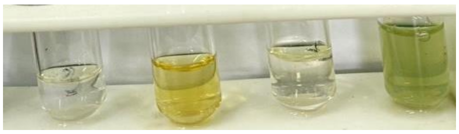	YJ-6	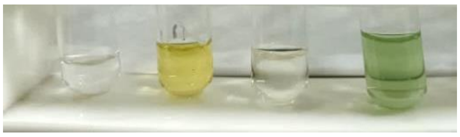

YJ-7

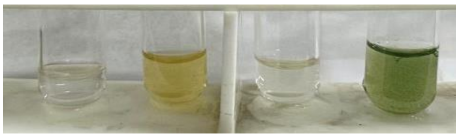

YJ-8

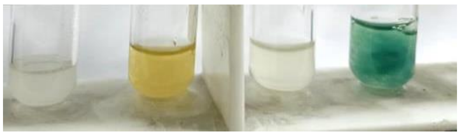

YJ-9

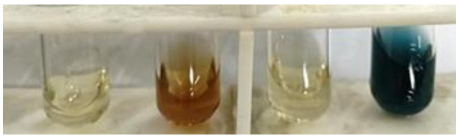

YJ-10

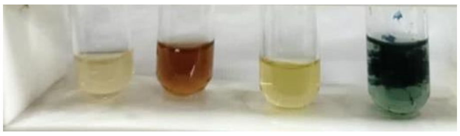

YJ-12

YJ-13

YG-1

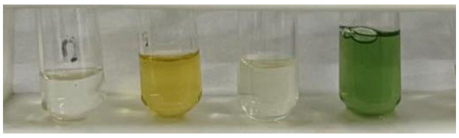

YG-2

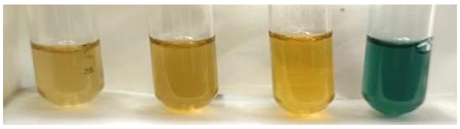

YG-4

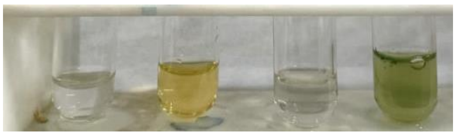

YG-5

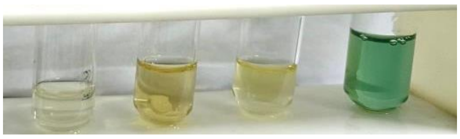

YG-6

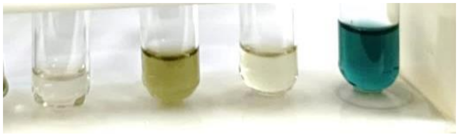

Supplementary Figure 3 Color reaction results of Flavonoids and Phenols

(A) The fermentation broth after fermentation; (B) The fermentation broth was reacted with 1% $FeCl_3$; (C) The fermentation broth was reacted with 1 mol/L NaOH; (D) The fermentation broth was reacted with 0.1% FeCl3:0.1% $K_3[Fe(CN)_6]$ = 1:1
