# Supplementary material for: Diversity, chemical constituents and biological activities of endophytic fungi from Alisma orientale (Sam.) Juzep
Source: Front Microbiol. 2023 Jun 21;14:1190624. doi: 10.3389/fmicb.2023.1190624 (PMC10320293; doi:10.3389/fmicb.2023.1190624)
Supplement: Supplementary file 5 [file Image_4.PDF]

Supplementary Material

Diversity, chemical constituents and biological activities of Endophytic fungi from *Alisma orientale* (Sam.) Juzep.

Nayu Shen[1]†, Zhao Chen[2]†, GuiXin Cheng[1]†, Wenjie Lin[1], Yihan Qin[1], Yirong Xiao[3], Hui Chen[1], Zizhong Tang[1*], Qingfeng Li[1], Ming Yuan[1], Tongliang Bu[1]

*** Correspondence:** Zizhong Tang**:** 14126@sicau.edu.cn

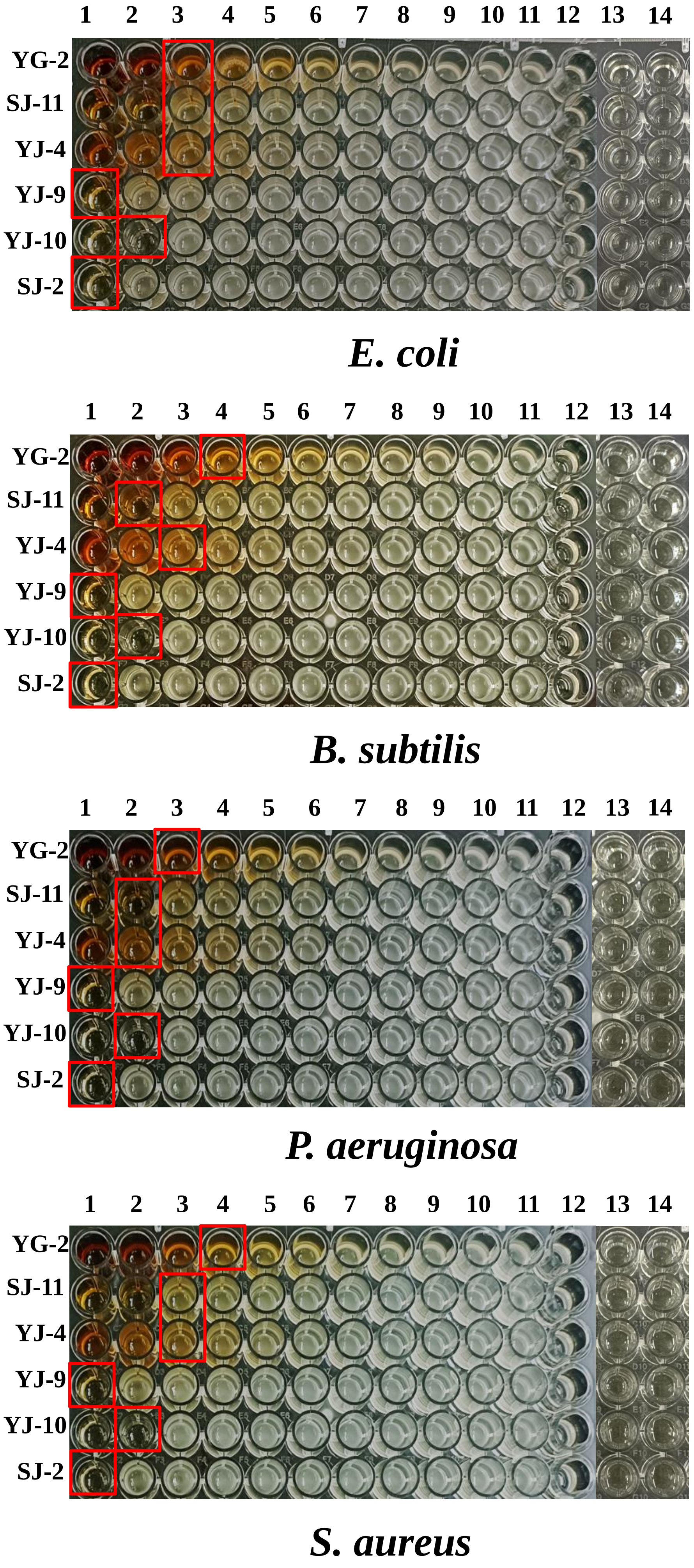
1 2 3 4 5 6 7 8 9 10 11 12 13 14
YG-2
SJ-11
YJ-4
YJ-9
YJ-10
SJ-2
E. coli
1 2 3 4 5 6 7 8 9 10 11 12 13 14
YG-2
SJ-11
YJ-4
YJ-9
YJ-10
SJ-2
B. subtilis
1 2 3 4 5 6 7 8 9 10 11 12 13 14
YG-2
SJ-11
YJ-4
YJ-9
YJ-10
SJ-2
P. aeruginosa
1 2 3 4 5 6 7 8 9 10 11 12 13 14
YG-2
SJ-11
YJ-4
YJ-9
YJ-10
SJ-2
S. aureus

Supplementary Figure 4 MIC of crude extracts against *E. coli, B. subtilis, P. aeruginosa* and *S. aureus*.

1-9: 5, 2.5, 1.25, 0.625, 0.313, 0.156, 0.078, 0.039, 0.0195 mg/mL crude extract solution; 10: Liquid LB containing bacterial suspension; 11: 5% DMSO solution containing bacterial suspension; 12: Liquid LB without bacterial suspension; 13: Liquid LB with 100 μg/mL ampicillin sodium; 14: Liquid LB with 100 μg/mL streptomycin sulphate.
