# Supplementary material for: Diversity, chemical constituents and biological activities of endophytic fungi from Alisma orientale (Sam.) Juzep
Source: Front Microbiol. 2023 Jun 21;14:1190624. doi: 10.3389/fmicb.2023.1190624 (PMC10320293; doi:10.3389/fmicb.2023.1190624)
Supplement: Supplementary file 6 [file Image_5.PDF]

Supplementary Material

Diversity, chemical constituents and biological activities of Endophytic fungi from *Alisma orientale* (Sam.) Juzep.

Nayu Shen[1]†, Zhao Chen[2]†, GuiXin Cheng[1]†, Wenjie Lin[1], Yihan Qin[1], Yirong Xiao[3], Hui Chen[1], Zizhong Tang[1*], Qingfeng Li[1], Ming Yuan[1], Tongliang Bu[1]

*** Correspondence:** Zizhong Tang**:** 14126@sicau.edu.cn

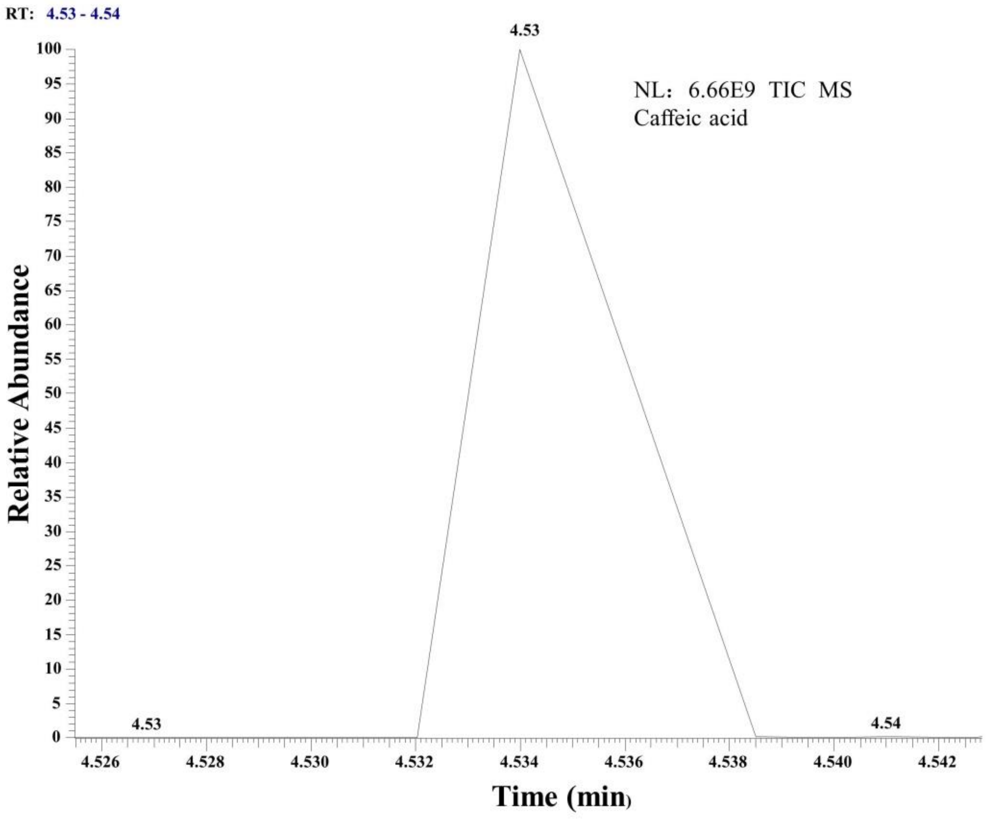
RT: 4.53 - 4.54
NL: 6.66E9 TIC MS
Caffeic acid
Relative Abundance
Time (min)

(A)

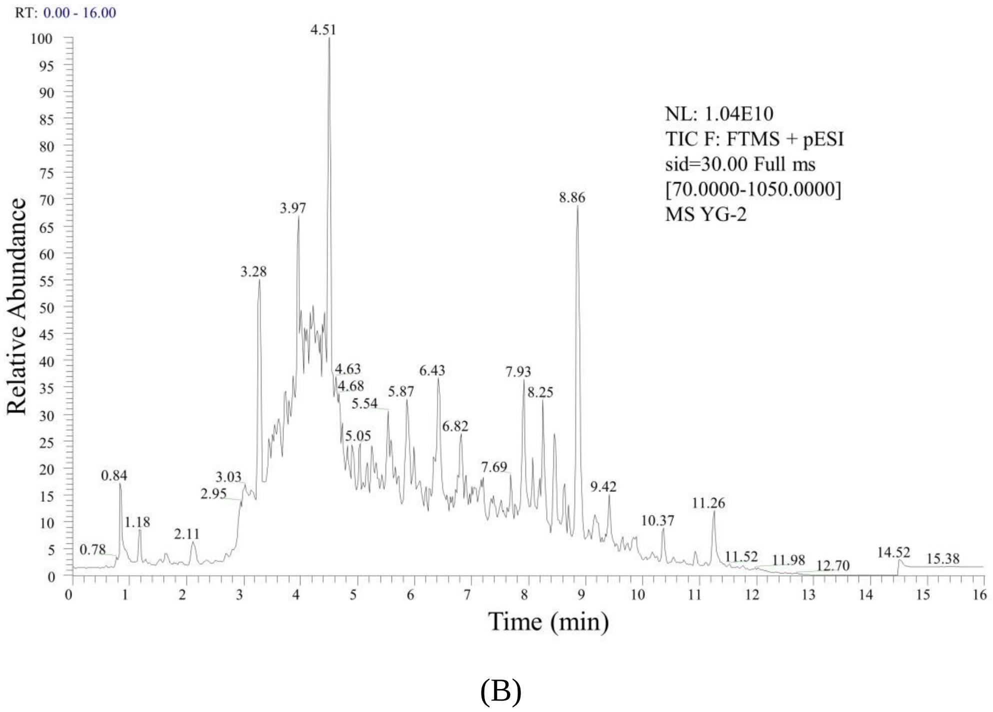
RT: 0.00 - 16.00
NL: 1.04E10
TIC F: FTMS + pESI
sid=30.00 Full ms
[70.0000-1050.0000]
MS YG-2
Relative Abundance
Time (min)

(B)

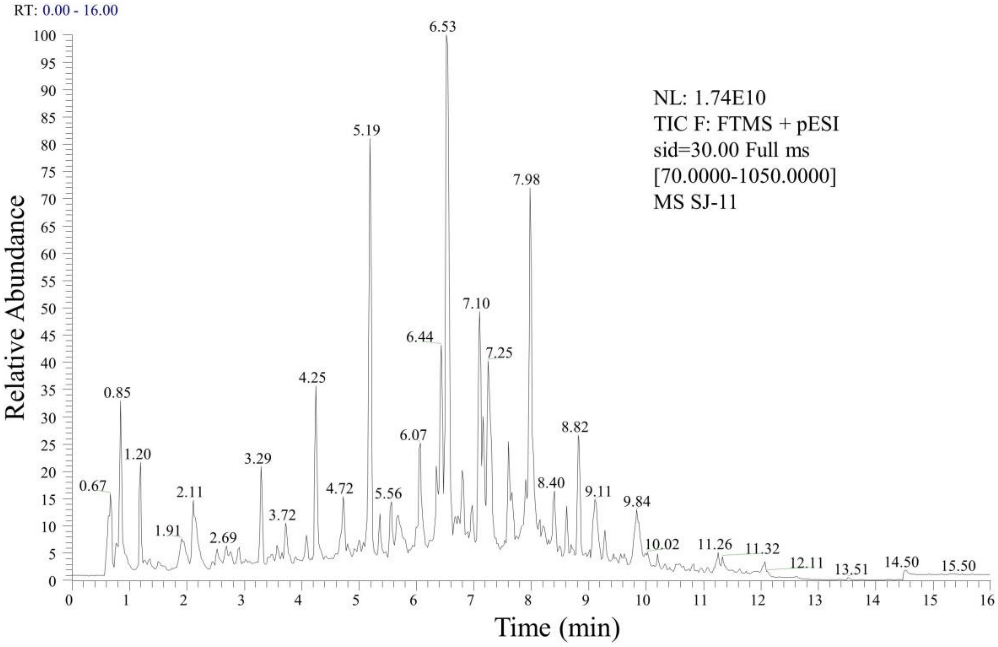
RT: 0.00 - 16.00
NL: 1.74E10
TIC F: FTMS + pESI
sid=30.00 Full ms
[70.0000-1050.0000]
MS SJ-11
Relative Abundance
Time (min)
6.53
5.19
7.98
7.10
6.44
7.25
4.25
0.85
1.20
3.29
6.07
8.82
0.67
2.11
8.40
9.11
9.84
4.72
5.56
3.72
1.91
2.69
10.02
11.26
11.32
12.11
13.51
14.50
15.50

(C)

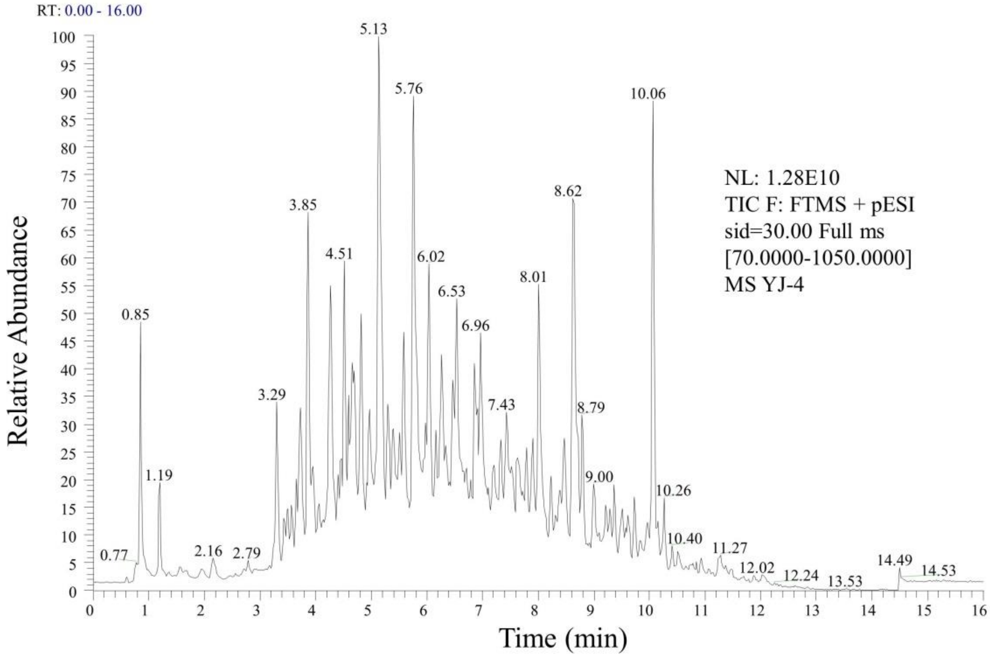
RT: 0.00 - 16.00
NL: 1.28E10
TIC F: FTMS + pESI
sid=30.00 Full ms
[70.0000-1050.0000]
MS YJ-4
Relative Abundance
Time (min)
5.13
5.76
10.06
3.85
8.62
4.51
6.02
6.53
8.01
0.85
6.96
3.29
7.43
8.79
1.19
9.00
10.26
10.40
11.27
0.77
2.16
2.79
12.02
12.24
13.53
14.49
14.53

（D）

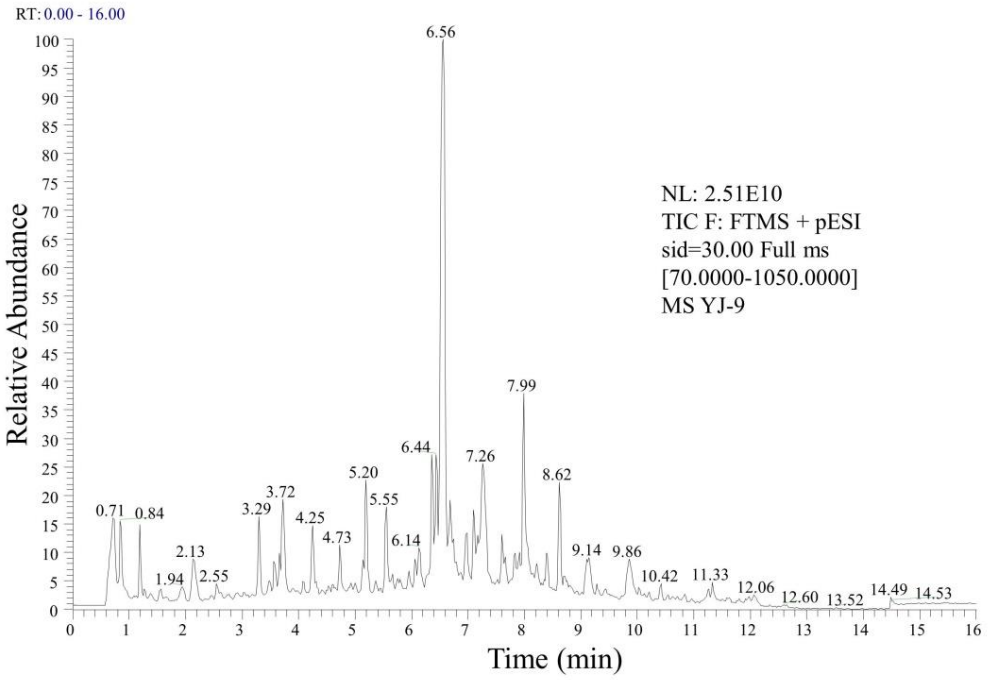

（E）

Supplementary Figure 5 Chromatogram.

(A) The LC-MS peak of Caffeic acid; (B) Crude extract of YG-2; (C) Crude extract of SJ-11; (D) Crude extract of YJ-4; (E) Crude extract of YJ-9.
