# Supplementary material for: Diversity, chemical constituents and biological activities of endophytic fungi from Alisma orientale (Sam.) Juzep
Source: Front Microbiol. 2023 Jun 21;14:1190624. doi: 10.3389/fmicb.2023.1190624 (PMC10320293; doi:10.3389/fmicb.2023.1190624)
Supplement: Supplementary file 7 [file Image_6.PDF]

Supplementary Material

Diversity, chemical constituents and biological activities of Endophytic fungi from *Alisma orientale* (Sam.) Juzep.

Nayu Shen[1]†, Zhao Chen[2]†, GuiXin Cheng[1]†, Wenjie Lin[1], Yihan Qin[1], Yirong Xiao[3], Hui Chen[1], Zizhong Tang[1*], Qingfeng Li[1], Ming Yuan[1], Tongliang Bu[1]

*** Correspondence:** Zizhong Tang**:** 14126@sicau.edu.cn

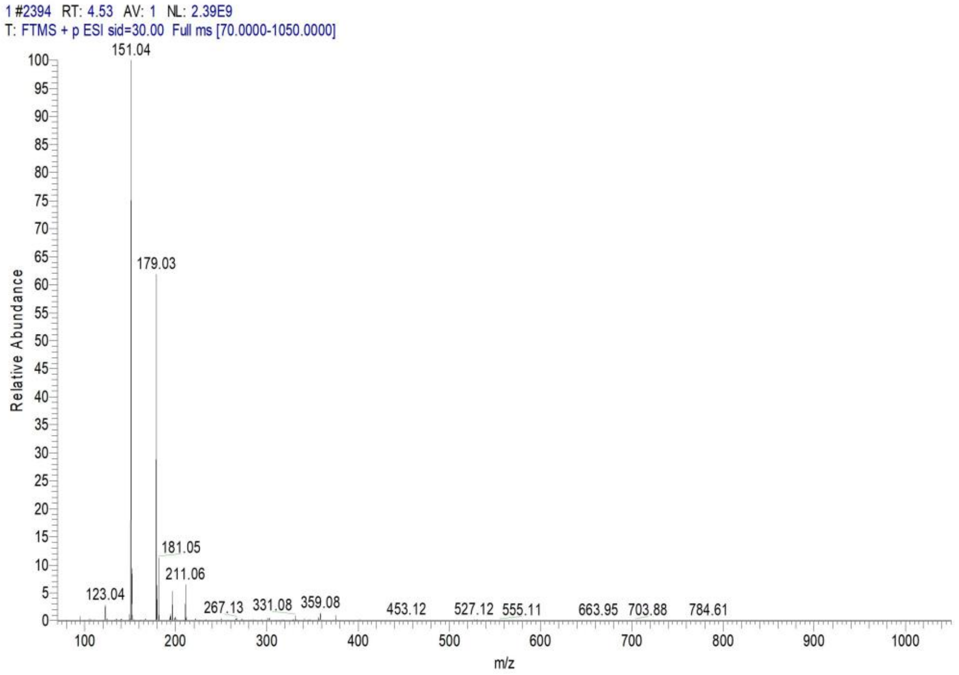
1 #2394 RT: 4.53 AV: 1 NL: 2.39E9
T: FTMS + p ESI sid=30.00 Full ms [70.0000-1050.0000]
151.04
179.03
181.05
211.06
123.04
267.13
331.08
359.08
453.12
527.12
555.11
663.95
703.88
784.61
Relative Abundance
m/z

(A)

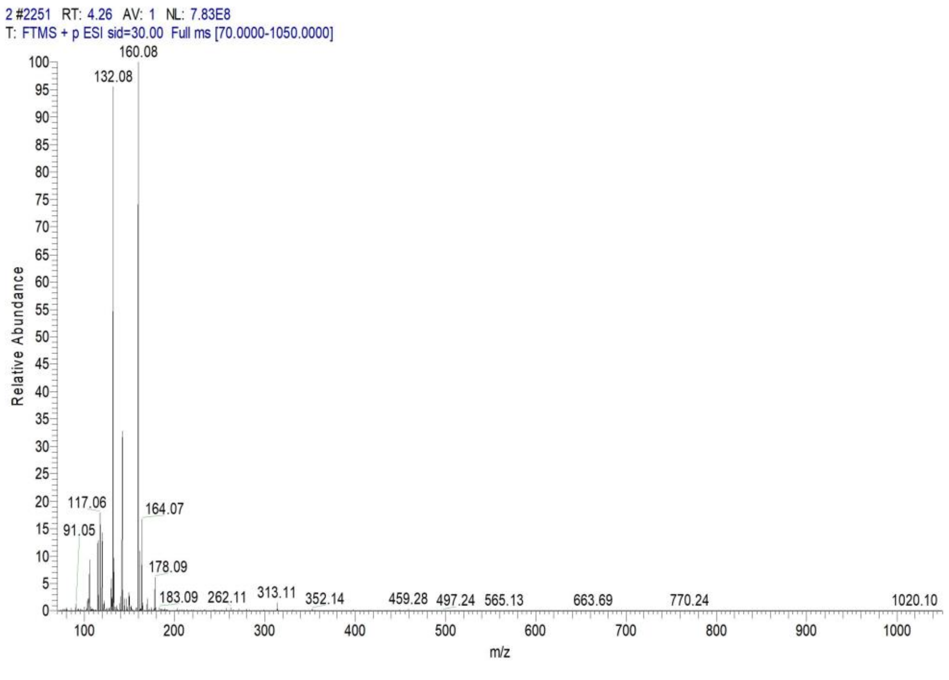
2 #2251 RT: 4.26 AV: 1 NL: 7.83E8
T: FTMS + p ESI sid=30.00 Full ms [70.0000-1050.0000]
160.08
132.08
117.06
91.05
164.07
178.09
183.09
262.11
313.11
352.14
459.28
497.24
565.13
663.69
770.24
1020.10
Relative Abundance
m/z

(B)

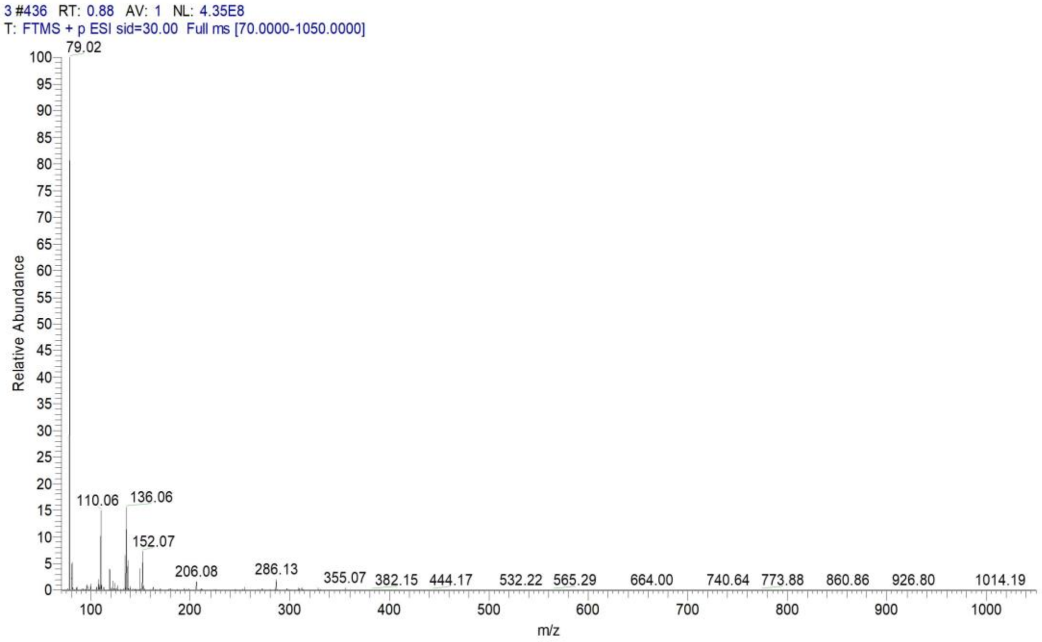

(C)

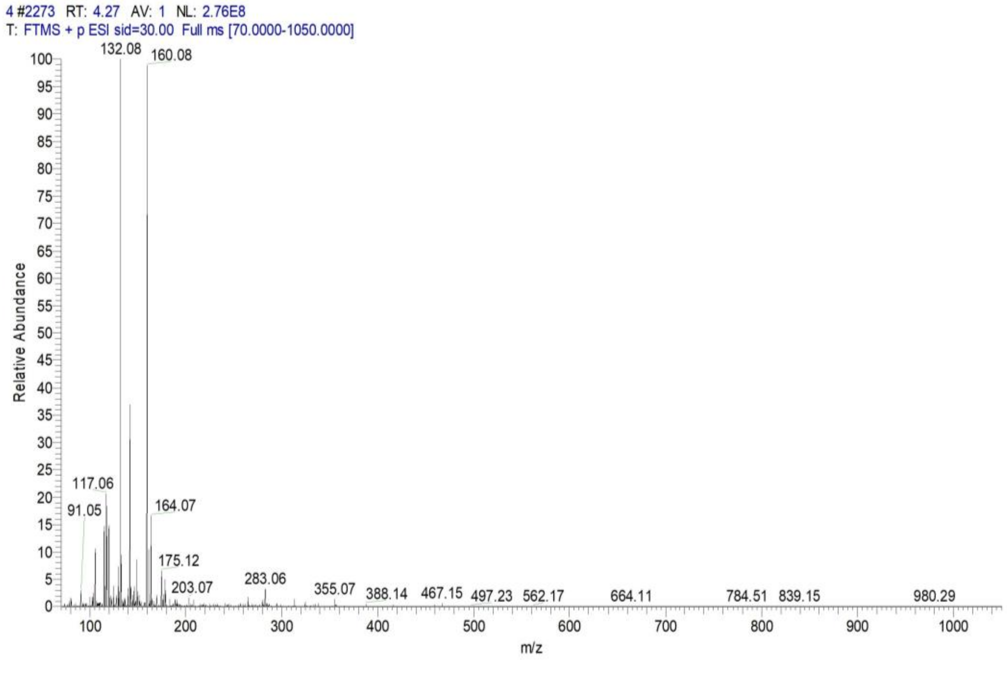

(D)

Supplementary Figure 6 Mass spectrum

(A) Caffeic aicd in YG-2; (B) Skatole in SJ-11; (C) Adenine in YJ-4; (D) Skatole in YJ-9.
